# Recyclable and air-stable colloidal manganese nanoparticles catalyzed hydrosilylation of alkenes with tertiary silane†

**DOI:** 10.1039/d4ra08380f

**Published:** 2025-01-20

**Authors:** Nobuki Katayama, Kazuki Tabaru, Tatsuki Nagata, Miku Yamaguchi, Takeyuki Suzuki, Takashi Toyao, Yuan Jing, Zen Maeno, Ken-ichi Shimizu, Takeshi Watanabe, Yasushi Obora

**Affiliations:** a Department of Chemistry and Materials Engineering, Faculty of Chemistry, Materials, and Bioengineering, Kansai University Suita Osaka 564-8680 Japan obora@kansai-u.ac.jp; b Comprehensive Analysis Centre, SANKEN, The University of Osaka Osaka 567-0047 Ibaraki Japan; c Institute for Catalysis, Hokkaido University Sapporo Hokkaido 001-0021 Japan; d School of Advanced Engineering, Kogakuin University Hachioji Tokyo 192-0015 Japan; e Japan Synchrotron Radiation Research Institute Sayo-gun Hyogo 679-5198 Japan

## Abstract

We synthesized *N*,*N*-dimethylformamide (DMF)-stabilized manganese nanoparticles (Mn NPs) in a one-step process under air using manganese(ii) chloride as the precursor. The Mn NPs were characterized in terms of particle size, oxidation state, and local structure using annular dark-field scanning transmission electron microscopy (ADF-STEM), X-ray photoelectron spectroscopy (XPS), and X-ray absorption spectroscopy (XAS). The results indicate that Mn NPs are divalent nanosized particles with Mn–O bonds. The Mn NPs exhibited high catalytic activity, achieving a turnover number (TON) of 15 800, surpassing previous manganese catalysts in alkene hydrosilylation. Furthermore, the Mn NPs maintained their catalytic activity after the reaction, enabling multiple recycling.

## Abstract 2

The manganese nanoparticles we synthesized demonstrated high catalytic activity, achieving 15 800 TON in the hydrosilylation of alkenes with tertiary silanes, and remained recyclable across multiple cycles.

## Graphical abstract

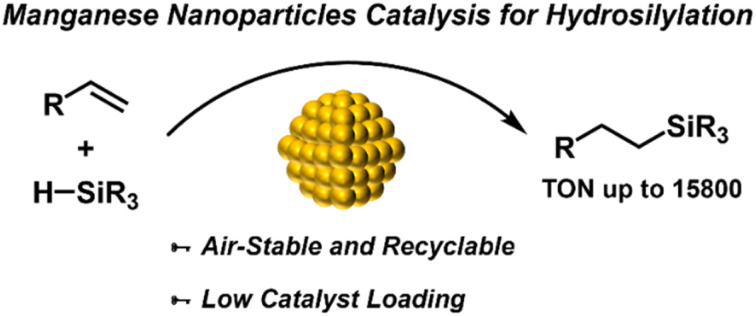

## Introduction

**Fig. 1:**
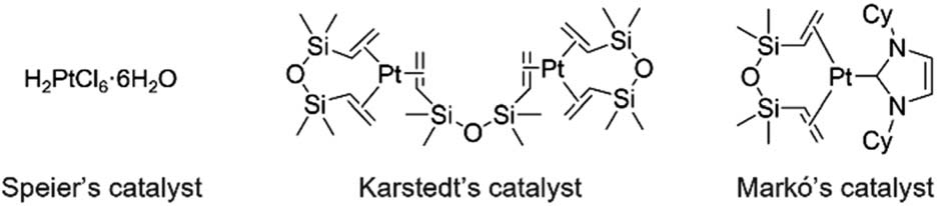
Platinum complex catalyst for hydrosilylation.

Hydrosilylation is essential for the synthesis of organosilicon compounds, which are used in numerous fields including organic synthesis, medicine, and industry.^1^ In general, precious-metal catalysts such as platinum complexes (Speier's,^2^ Karstedt's,^3^ and Markó's^4^ catalysts) are used for this reaction. However, these metals are rare, toxic, and costly, necessitating the development of alternative catalysts (Fig. 1).^5^

**Scheme 1:**
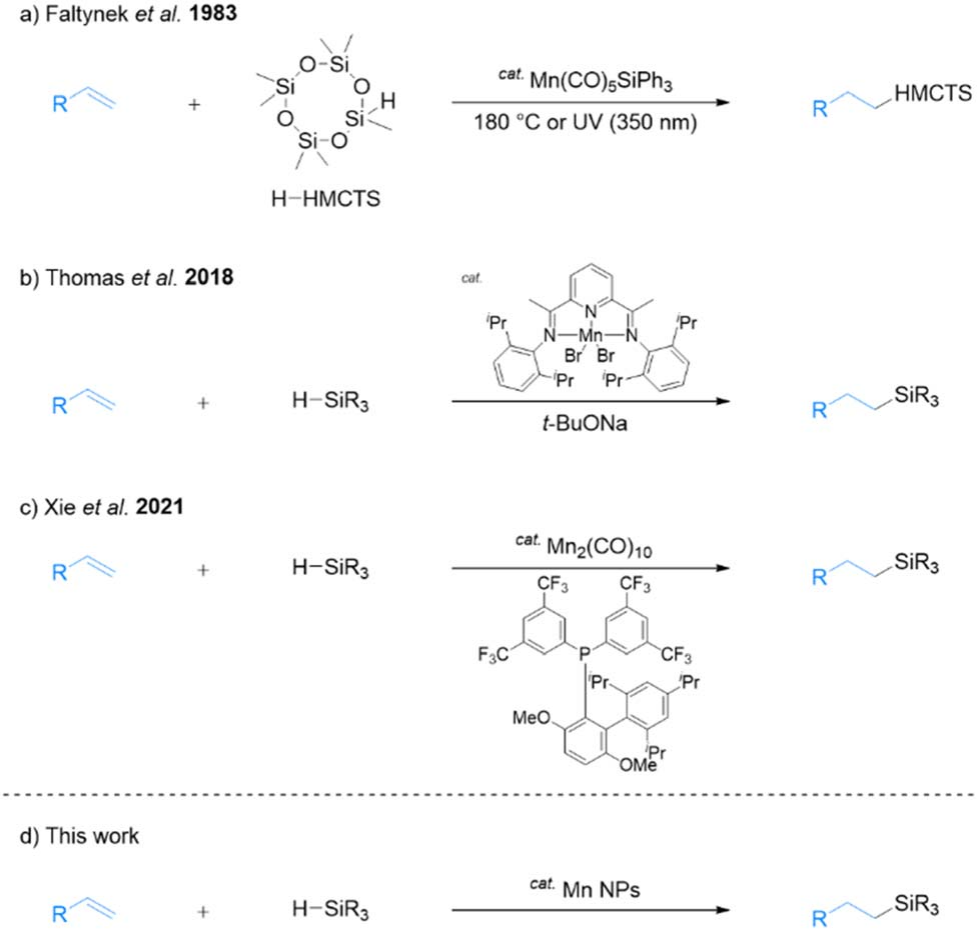
Manganese-catalyzed hydosilylation.

In recent years, the utility of earth-abundant first-row-transition-metal and non-noble-metal catalysts has attracted widespread research attention in the chemical sciences community. These investigations have primarily focused on metals such as iron,^6^ cobalt,^7^ and nickel^8^ for hydrosilylation. Manganese is the third most abundant transition metal in Earth's crust and is considered non-toxic and environmentally friendly.^9^ Manganese is an attractive candidate as a catalyst for a wide range of organic reactions^10^ and is useful as a catalyst for alkene hydrosilylation.^11^ In one early study, Faltynek successfully catalyzed hydrosilylation using a triphenylsilyl manganese complex. The reaction involved heptamethylcyclotetrasiloxane (H-HMCTS) and 1-pentene as substrates and was carried out by heating at 180 °C or with UV irradiation at 350 nm (Scheme 1a).^12^ Later, Thomas's group reported that the combination of a bis(imino)pyridine manganese complex and sodium *tert*-butoxide exhibited high catalytic activity for hydrosilylation of alkenes with tertiary silanes (Scheme 1b).^13^ Sodium *tert*-butoxide is an additive with good catalytic activity, but it is known to react with alkoxides and alkoxysilanes to form flammable SiH_4_.^14^ Therefore, challenges remain in the selection of practical additives.

Recently, Xie and co-workers reported that Mn_2_(CO)_10_ tuned by the electron-deficient JackiePhos ligand catalyzes the hydrosilylation of alkenes (Scheme 1c).^15^ In their study, the addition of 2,2,6,6-tetramethylpiperidin-1-oxyl (TEMPO) free radicals almost completely inhibited the reaction, suggesting that a radical mechanism is involved. Specifically, the thermal dissociation of ligand-assisted Mn_2_(CO)_10_ generates a manganese-centered radical that subsequently generates a silyl radical that adds to the alkene. Finally, hydrogen atom transfer occurs from the manganese hydride produces the hydrosilylated product. This reaction also showed high catalytic activity for dehydrosilylation upon modifying the ligand. However, these homogeneous catalysts are difficult to recover from the hydrosilylated products, necessitating the development of recyclable heterogeneous catalysts with low environmental impact. Accordingly, the present study focuses on exploring the potential of transition-metal nanoparticles (NPs) as an alternative.

Transition-metal NPs exhibit high catalytic activities, even at low catalyst loadings, owing to their larger surface areas compared with those of the bulk metals.^16^ NPs exhibit catalytic activities that are different from those of conventional molecular-complex and heterogeneous catalysts, and their physical properties and application to numerous catalytic reactions are an active area of study. Although there are various methods for synthesizing metal NPs, we have focused on the liquid-phase synthesis method. Several methods for synthesizing Mn NPs by liquid-phase synthesis have been reported, but they have not yet been applied catalytically.^17^

Our group reported that various metal NPs synthesized by a one-step liquid-phase synthesis using *N*,*N*-dimethylformamide (DMF)^18^ exhibited high catalytic activity in organic reactions.^19^ Specifically, DMF-stabilized Fe_2_O_3_ NPs exhibit high catalytic activity for the hydrosilylation of alkenes with primary and secondary silanes, resulting in the formation of hydrosilylated products without additives.^20^ However, DMF-stabilized Fe_2_O_3_ NPs are not effective for hydrosilylation with tertiary silanes. To address this limitation, we reported that a DMF-stabilized combination of Fe_2_O_3_ NPs with Pt NPs can catalyze the *anti*-Markovnikov selective hydrosilylation of alkenes with tertiary silanes.^21^ While this combination represents a significant improvement of our methodology, it requires the use of Pt. Accordingly, we synthesized manganese NPs using DMF reduction and explored their potential as an effective Pt-free catalyst for hydrosilylation with tertiary silanes (Scheme 1d). The use of DMF-stabilized Mn NPs presents a more cost-effective and sustainable method for catalyzing hydrosilylation reactions, and they can be easily recycled multiple times following a simple extraction process. The present report includes details of the structural characterization of Mn NPs and their application to the hydrosilylation of alkenes with tertiary silanes.

## Results and discussion

**Scheme 2:**
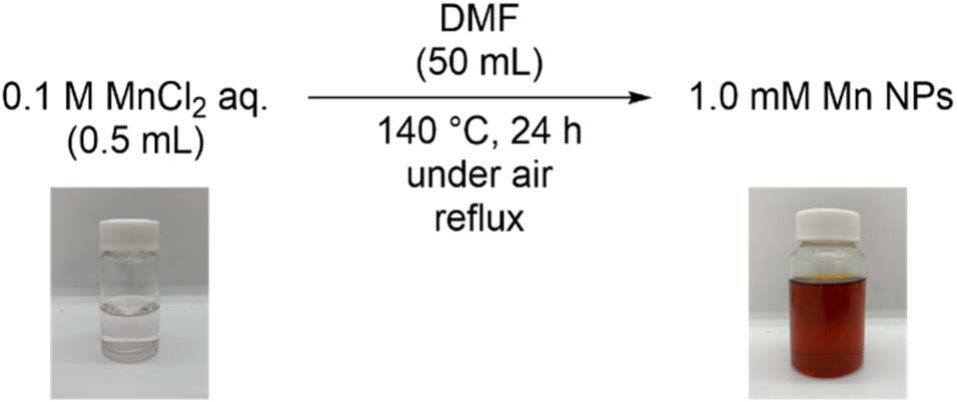
Synthesis of DMF-stabilized manganese nanoparticles.

**Fig. 2:**
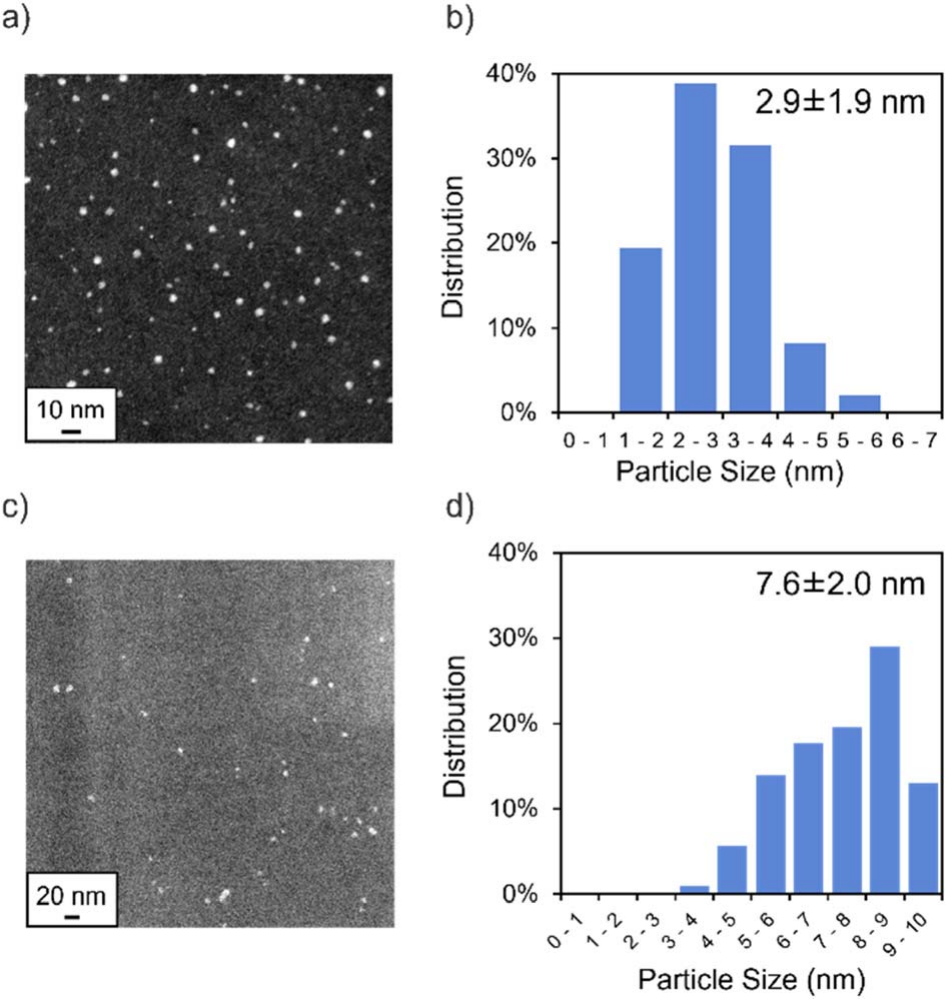
(a) ADF-STEM image of the Mn NPs before reaction (scale bar = 10 nm) (b) size distribution of the Mn NPs before reaction (c) ADF-STEM image of the Mn NPs after reaction (scale bar = 20 nm) (d) size distribution of the Mn NPs after reaction.

We synthesized DMF-stabilized Mn NPs from aqueous manganese chloride solution by heating and stirring at 140 °C with a reflux condenser in DMF (Scheme 2 and see ESI† for details). Annular dark-field scanning transmission electron microscopy (ADF-STEM; Fig. 2) and energy dispersive X-ray spectroscopy (EDX; Fig. S8†) show that the average particle size of the Mn NPs before and after hydrosilylation reaction are 2.9 and 7.6 nm, respectively. Thus, the Mn NPs exhibit a significant size increase over the course of hydrosilylation reaction, yet they remain within the nanoscale size range. The coordination state of the DMF layer surrounding the DMF-stabilized Mn NPs was investigated by Fourier transform infrared spectroscopy (FT-IR; Fig. S9†). The C=O and O–C–N vibrational modes of DMF in the Mn NPs were observed to shift relative to the corresponding peaks of DMF molecules. In particular, the strong absorption peak at 1675 cm^−1^, attributed to the C=O vibration of DMF molecules, shifted to lower wavenumbers of 1672 cm^−1^ for Mn NPs_br and 1664 cm^−1^ for Mn NPs_ar indicating an interaction between the C=O groups in DMF and the Mn NPs. Similarly, the O–C–N band of DMF, initially at 658 cm^−1^, shifted to 660 cm^−1^ for Mn NPs_br and to 662 cm^−1^ for Mn NPs_ar. These shifts suggest that the amide groups of DMF interact with the Mn NPs. In addition, Mn NPs_ar showed a peak in the range of 700–900 cm^−1^, which was not present in Mn NPs_br. These peaks indicate the presence of silane compounds in Mn NPs_ar. The incorporation of these compounds is probably related to the increased particle size of Mn NPs after the hydrosilylation reaction. X-ray diffraction (XRD) analysis could not be performed due to the ultra-small size of the sample (Fig. S10†).

**Fig. 3:**
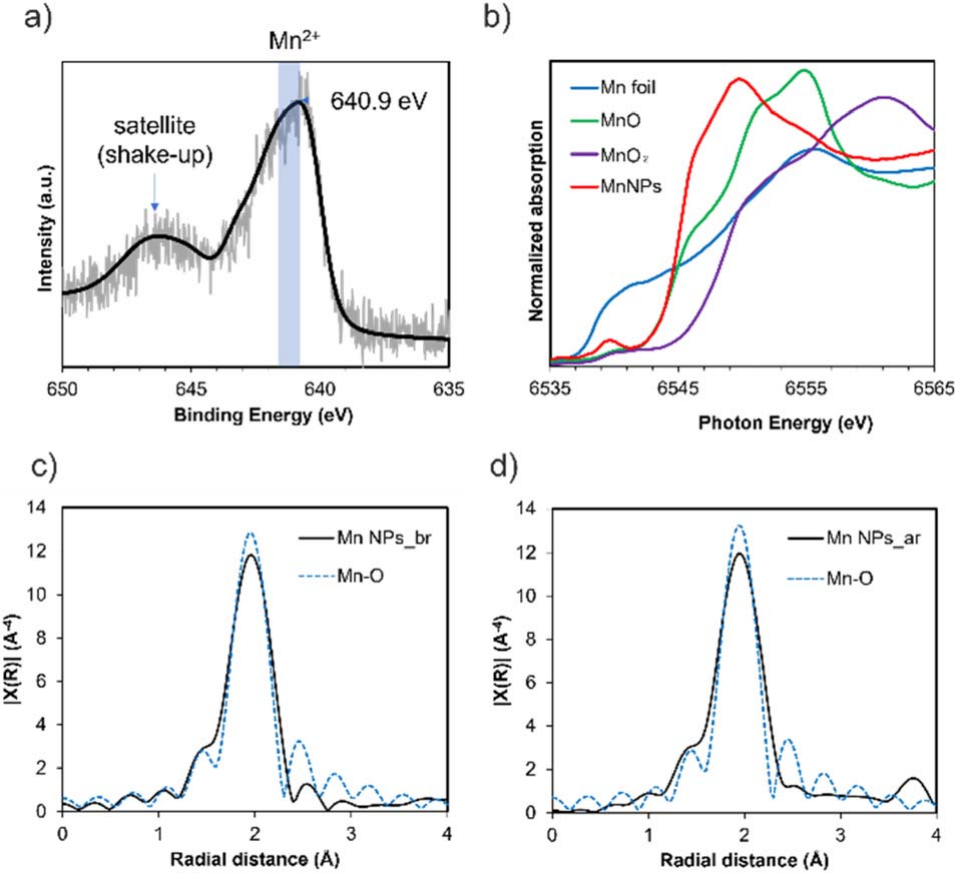
(a) XPS profiles for the Mn 2p_3/2_ region for Mn NPs and (b) Mn K-edge XANES spectra of the reference Mn compounds and Mn NPs, and (c) Fourier-transformed *k*^3^-weighted Mn K-edge EXAFS spectra of Mn NPs_before reaction (Mn NPs_br) and (d) after reaction (Mn NPs_ar).

The oxidation state of the Mn NPs was analyzed by X-ray photoelectron spectroscopy (XPS) and X-ray absorption spectroscopy (XAS). In the Mn 2p XPS spectrum of the DMF-stabilized Mn NPs, the Mn 2p_3/2_ peak appears at 640.9 eV (Fig. 3a). The Mn^2+^ peak for the Mn 2p_3/2_ region has been reported to occur at 640.8–641.6 eV.^17,22^ Furthermore, satellite peaks (shake-up) for MnO (Mn^2+^) are observed. These suggest that the DMF-stabilized Mn NPs are divalent.

X-ray absorption near edge structure (XANES) analysis of the DMF-stabilized Mn NPs was conducted, and the results were compared with those for Mn(0) foil, MnO pellets, and MnO_2_ pellets. The position of the absorption edge of the Mn NPs is similar to that of MnO (*E*_0_: Mn NPs = 6545.2 eV, MnO = 6545.1 eV), indicating that they are divalent (Fig. 3b). *E*_0_ was calculated by the local maximum of the first derivative of the XANES spectrum (d*μ*/d*E*). This result is consistent with the XPS results and indicates that the oxidation state of the Mn NPs is divalent. The *E*_0_ of 6545.2 eV for Mn NPs after the reaction suggests that the Mn NPs remain divalent throughout the reaction (Fig. S6†). There is a slight difference in the XANES spectra shapes of Mn NPs and MnO. This can be attributed to the liquid form of the XAS sample of Mn NPs and the presence of chloride ions in the precursor (Fig. S7†). In addition, the XPS spectrum of Mn NPs revealed a chloride peak, suggesting the presence of residual chloride species (Fig. S3†).

Next, the extended X-ray absorption fine structure (EXAFS) spectra were Fourier transformed into radial space (R-space) to analyze the local structure around the Mn NPs (Fig. 3c and d). The results show that before the reaction, the results for Mn NPs can be fitted to Mn–O bonds with a bond distance of 2.26 Å and a coordination number of 4.1 (Table S2†). After the reaction, Mn–O bonds distance of 2.25 Å and coordination number 4.0 were found to best fit the results. This suggests that Mn–O bonds are present before and after the reaction and that the coordination atom species around the Mn atom are the same in the Mn NPs before and after the reaction. This indicated that the Mn NPs would be recyclable. To summarize the XPS and XAS analysis, the XPS and XANES results indicate that the oxidation state of the Mn NPs is divalent and that residual chloride ions are present, whereas the EXAFS results confirm that the structure of the Mn NPs consists of Mn–O bonds.

We investigated the Mn NPs-catalyzed hydrosilylation of 1-dodecene (1a: 0.5 mmol) with methyldiphenylsilane (2a: 2.5 mmol) as model substrates under various conditions (Table 1). For instance, the reaction of 1a with 2a in the presence of Mn NPs (0.01 mol%) at 130 °C for 24 h gave the main product 3a in 70% yield (entry 1). GC and GC-MS analysis confirmed that no by-products such as Markovnikov or vinylsilane products were formed in this catalytic reaction. Performing the reaction without a catalyst confirmed that the catalyst is required for this reaction to proceed (entry 2). Upon lowering the temperature to 100 °C, the reaction did not proceed and no hydrosilylated product was obtained (entry 3). The use of diglyme, a high-boiling-point ether solvent, improved the yield of 3a (entry 4). This improvement is attributed to the high dispersity of the Mn NPs in the solvent. Since this Mn NPs has Mn–O bonds, MnO with these bonds were used as catalysts for the reaction. As a result, the corresponding product was not observed with MnO because the substrate was hardly converted (entry 5). A key difference between Mn NPs and MnO is their dispersibility in the reaction solution: MnO is insoluble, while Mn NPs are highly dispersed in the solution. This clear difference contributes to the catalytic activity of Mn NPs in hydrosilylation. The high dispersion of Mn NPs in the solution is thought to facilitate reactions with the substrate, leading to the generation of active species, such as silyl radicals, which subsequently lead to the formation of the desired product. When the model reaction was performed in the presence of a catalytic amount of TEMPO or 3,5-di-*tert*-butyl-4-hydroxytoluene (BHT) as a radical scavenger, hydrosilylation was inhibited (entry 6 and Table S6†). Moreover, the silyl radical adduct was detected by HRMS when TEMPO was added, indicating that silyl radicals are the active species in the reaction (Fig. S11, S12 and S15†). Since residual chlorine ions were observed in the Mn NPs, the reaction was carried out using HCl as the acid catalyst (Table S7,† entry 3). As a result, although the conversion was high, isomerization of alkenes and disproportionation of hydrosilanes occurred, resulting in low yields.

**Table 1:** Optimization of Mn NPs-catalyzed hydrosilylation of 1-dodecene (1a) with methyldiphenylsilane (2a)^a^

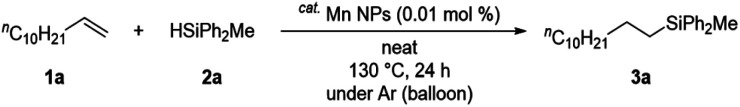		
Entry	Reaction conditions	Yield^b^^c^ (%)
1	Standard condition	70
2	In the absence of Mn NPs	nd
3	100 °C	nd
4	Diglyme (1 mL) as the solvent	86 [83]^d^
5	MnO (20 mol%) instead of Mn NPs	nd
6	TEMPO (20 mol%)	nd

a Reaction conditions: 1-dodecene 1a (0.5 mmol), methyldiphenylsilane 2a (2.5 mmol) and Mn NPs (0.01 mol%) at 130 °C for 24 h under Ar atmosphere.

b Yields were determined by GC based on 1a used (*n*-nonane as internal standard).

c nd = not detected by GC.

d Number in square brackets show the isolated yield.

The substrate scope of the alkene hydrosilylation was investigated with various alkenes and hydrosilanes (Table 2). Aliphatic alkenes and allylbenzenes gave the corresponding products in good yields (3a–f). Oxygen-containing functional alkenes, such as ketones, esters, epoxides, and alcohol, were well-tolerated in the reaction, yielding the corresponding products (3g–n) with good to moderate yields. In addition, in the presence of carbonyl derivatives, the reaction selectively proceeded towards the alkene hydrosilylation products (3g–i), while exhibiting no reactivity towards the C=O groups. The desired product was obtained even when cyclooctene, an internal alkene, was used (3o). The screening of other hydrosilanes, such as HSiMe_2_Ph, a secondary silane (H_2_SiPh_2_), and a primary silane (H_3_SiPh), demonstrated their amenability to the reaction system, affording the desired products in good to excellent yields (3p–r).

**Table 2:** Substrate scope of Mn NPs-catalyzed hydrosilylation^a^

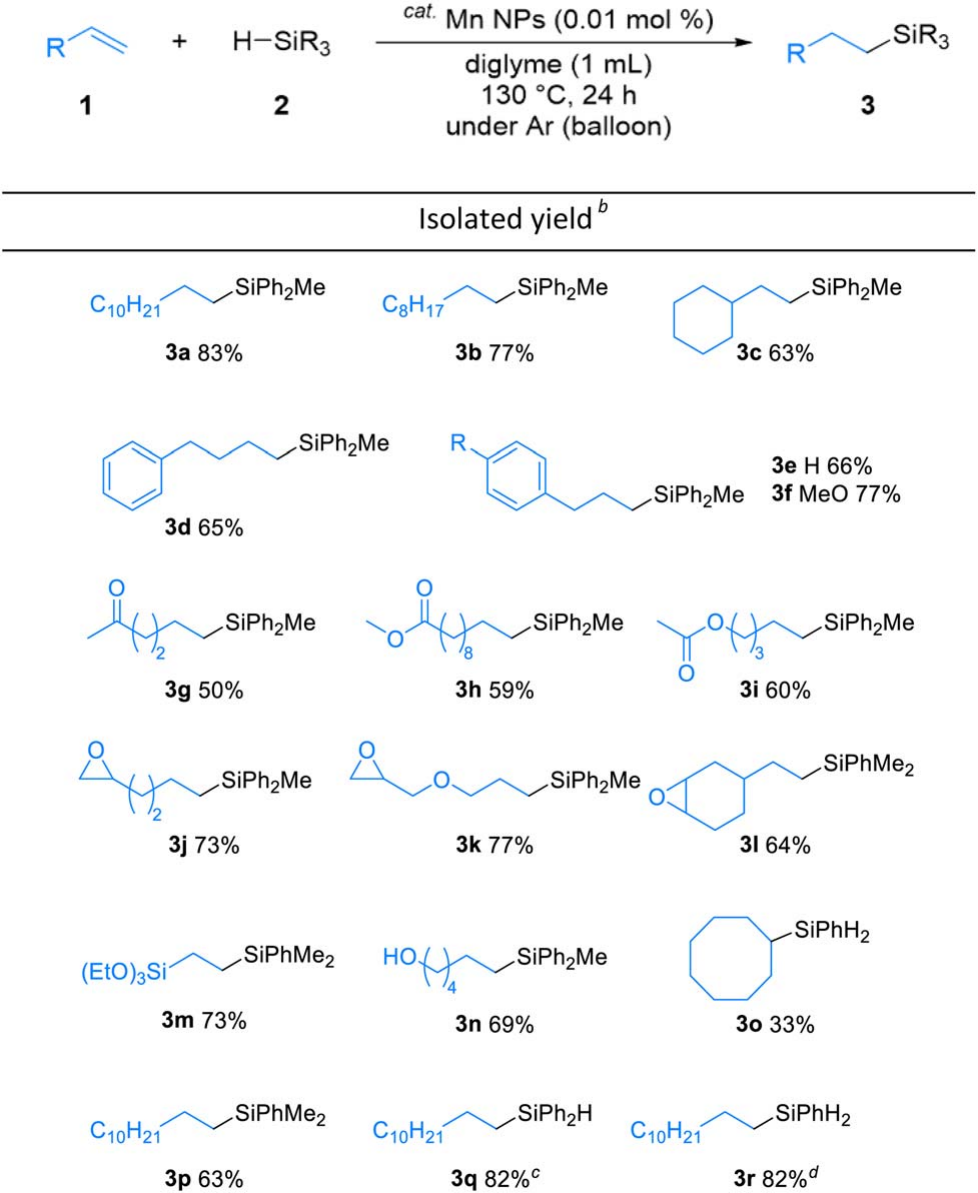

a Reaction conditions: alkenes 1 (0.5 mmol), hydrosilanes 2 (2.5 mmol) and Mn NPs (0.01 mol%) in 1.0 mL diglyme at 130 °C for 24 h under Ar atmosphere, unless otherwise noted. The structure was identified by NMR measurement.

b Yields of isolated product after purification.

c Reaction was proceeded at 110 °C.

d Reaction was proceeded at 100 °C.

**Scheme 3:**
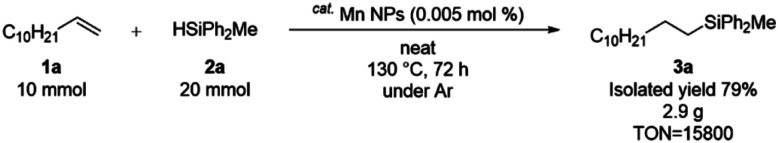
Multi-gram scale hydrosilylation.

To further demonstrate the synthetic utility of the Mn NPs catalyst, we conducted a multi-gram scale hydrosilylation (Scheme 3). The reaction of 1a (10 mmol) and 2b (20 mmol) afforded the hydrosilylated products in a yield of 79%. By scaling up, it was possible to reduce the substrate-to-hydrosilane ratio as well as decrease the catalyst amount to 0.005% (TON = 15 800). This TON represents the highest value reported to date for manganese-catalyzed hydrosilylation of alkenes (Scheme S3†).

**Fig. 4:**
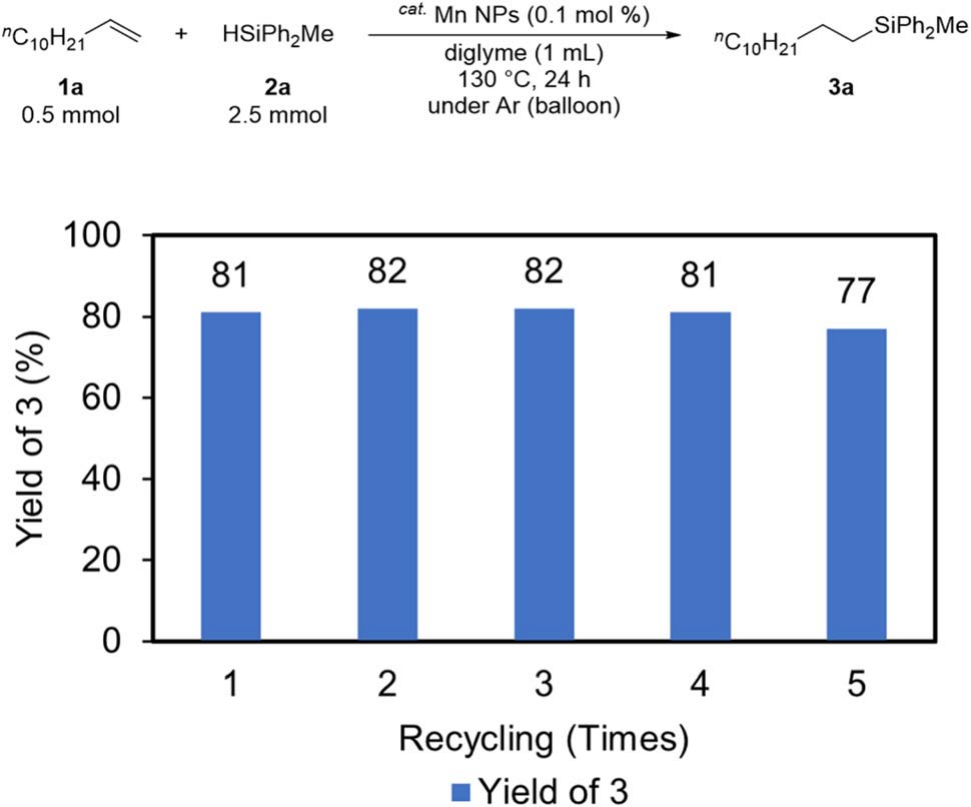
Recycling experiment result for Mn NPs-catalyzed hydrosilylation of 1a with 2a.

Recycling experiments were then conducted to evaluate the activity of the catalyst after the reaction. In the recycling procedure, hexane (8 mL) and DMF (2 mL) were added to the solution after the reaction, and the solution was separated into two layers (an upper hexane layer and lower DMF layer). The solution was then extracted five times with hexane (8 mL), recovering the substrate and product in the hexane layer and leaving the catalyst in the DMF layer. The recovered catalyst was recycled for subsequent hydrosilylation after DMF evaporation. The Mn NPs remained catalytically active, as the desired product was obtained in high yield even after five recycles (Fig. 4).

## Conclusions

In summary, we synthesized DMF-stabilized Mn NPs using a DMF-reduction method and characterized them as divalent Mn NPs with Mn–O bonds (1–5 nm) by annular dark-field scanning transmission electron microscope, energy dispersive X-ray, X-ray photoelectron spectroscopy, X-ray adsorption near-edge structure, and extended X-ray absorption fine structure analyses. The Mn NPs catalyzed hydrosilylation reactions in high yields and high product selectivities at low catalyst loading, and they were also found to be applicable to a wide variety of substrates and to be recyclable multiple times.

## Data availability

The data supporting this article have been included as part of the ESI.†

## Author contributions

N. K. conducted the experiments, performed the analysis, and wrote the manuscript. Y. O. supervised the work and edited the manuscript. T. N. and M. Y. contributed to the experimental work. N. K., K. T., T. N., T. T., Y. J., Z. M., K. S., and T. W. performed the XAS measurements and analyses. T. S. conducted the TEM and ICP analysis. The manuscript was written through contribution of all authors. All authors have given approval to the final version of the manuscript.

## Conflicts of interest

There are no conflicts to declare.

## Supplementary Material

RA-015-D4RA08380F-s001
