# Supplementary material for: Recyclable and air-stable colloidal manganese nanoparticles catalyzed hydrosilylation of alkenes with tertiary silane
Source: RSC Adv. 2025 Jan 20;15(3):1776–81. doi: 10.1039/d4ra08380f (PMC11744517; doi:10.1039/d4ra08380f)
Supplement: RA-015-D4RA08380F-s001 [file RA-015-D4RA08380F-s001.pdf]

Supplementary Information (SI) for RSC Advances.
This journal is © The Royal Society of Chemistry 2025

Electronic Supporting Information

Recyclable and air-stable colloidal manganese nanoparticles catalyzed hydrosilylation of alkene with tertiary silane

Nobuki Katayama,[a] Kazuki Tabaru,[a] Tatsuki Nagata,[a] Miku Yamaguchi,[a] Takeyuki Suzuki,[b] Yuan Jing,[c] Takashi Toyao,[c] Zen Maeno,[d] Ken-ichi Shimizu,[c] Takeshi Watanabe,[e] and Yasushi Obora*[a]

[a] *Department of Chemistry and Materials Engineering, Faculty of Chemistry, Materials and Bioengineering, Kansai University, Suita, Osaka 564-8680, Japan*

[b] *Comprehensive Analysis Centre, SANKEN, The University of Osaka, Ibaraki, Osaka 567-0047, Japan*

[c] *Institute for Catalysis, Hokkaido University, Sapporo, Hokkaido 001-0021, Japan*

[d] *School of Advanced Engineering, Kogakuin University, Hachioji, Tokyo 192-0015, Japan*

[e] *Japan Synchrotron Radiation Research Institute, Sayo-gun, Hyogo 679-5198, Japan*

* obora@kansai-u.ac.jp

Table of Contents

1 General information S2

2 Experimental procedures S3

3 Results of PL and UV-Vis spectroscopic analyses S5

4 Results of XPS analysis S6

5 Results of XAS analysis S8

6 Recycling experimental S9

7 Other information S10

8 Screening information S16

9 Characterization data S18

10 NMR-Spectra S27

11 References S42

1. General information

Gas chromatography (GC) was performed using a GC-2025 system (Shimadzu Co.) equipped with a flame ionization detector and a 0.22 mm × 25 m BP-5 capillary column. ^{1}H and ^{13}C nuclear magnetic resonance (NMR) spectra were acquired at 400 and 100 MHz, respectively, in $CDCl_3$ with tetramethylsilane as an internal standard (JNM-ECZ-400S, Japan Electron Optics Laboratory Co.). ^{1}H NMR chemical shifts were referenced to tetramethylsilane (0 ppm) or residual chloroform (7.26 ppm) as an internal standard. ^{13}C NMR chemical shifts were referenced to residual chloroform peaks (77 ppm). All substrates were commercially available and used without purification. Compounds **3b**,[1] **3d**,[2] **3g**,[3] **3l**,[4] **3m**,[5] **3o**,[6] **3q**,[7] and **3r**[8] were reported previously. The products were characterized by ^{1}H NMR and ^{13}C NMR spectroscopies, and GC-mass spectrometry (GC-MS, electron ionization). Compounds **3a**, **3c**, **3e**, **3f**, **3h**, **3i**, **3j**, **3k**, **3n**, and **3p** were also examined by Fourier transform infrared spectroscopy (FTIR, Shimadzu IRAffinity-1, Shimadzu Co.) and high-resolution MS (micrOTOF with ESI source, Bruker Inc.). GC-MS spectra were recorded with a GCMS-QP2010 SE instrument (Shimadzu Co.). The product yields were estimated from GC peak areas using the internal standard technique (*n*-nonane as the internal standard). X-ray photoelectron spectroscopy (XPS) data were acquired using a PHI5000 VersaProbe instrument (ULVAC-PHI, Inc.) with an Al Kα X-ray source. Annular dark-field scanning transmission electron microscopy (ADF-STEM) images were obtained using a JEM-ARM200F instrument (Japan Electron Optics Laboratory, Co.) at an accelerating voltage of 200 kV. The measurement range was 300–800 nm. Photoluminescence (PL) spectra were recorded with a spectrofluorometer (RF-6000, Shimadzu Co.), with an excitation wavelength of 350 nm. Fluorescence spectra were recorded with a data interval of 0.5 nm, covering a fluorescence wavelength range of 360–600 nm, at a scanning rate of 6000 nm/min. Ultraviolet-visible (UV-Vis) spectra were obtained using a UV-Vis spectrophotometer (UV-1900i, Shimadzu Co.). FTIR spectra were obtained using the liquid-film method on NaCl plates with an IRAffinity-1 FT-IR spectrometer (Shimadzu Co.) X-ray diffraction patterns were recorded using a MiniFlex600-C instrument (Rigaku). Mn K-edge X-ray absorption spectroscopy (XAS) was conducted in transmittance and fluorescence modes at the BL14B2 beamline (operated at 8 GeV) of SPring-8 (Hyogo, Japan) with a Si(111) double-crystal monochromator (proposal 2021A1615, 2022B1952).[9] Data analysis was performed using Athena software, included in the Demeter package.[10] The Fourier transformation of the k^3-weighted extended X-ray absorption fine structure (EXAFS) was performed over the k range 3–12 Å^{-1}.

2. Experimental procedures

Typical procedure for Mn NPs-catalyzed hydrosilylation of 1a with 2a (Table 1, entry 4)

Mn NPs (50 μL, 0.01 mol%) were added to a Schlenk tube and the DMF was removed under vacuum. Then 1-dodecene (**1a**: 0.5 mmol), methyldiphenylsilane (**2a**: 2.5 mmol) and diglyme (1 mL) as the solvent were added and the reaction was carried out at 130 °C under Ar conditions for 24 h. The conversions and product yields were estimated from GC peak areas based on an internal standard.

The desired product was isolated by column chromatography (silica gel, hexane/ethyl acetate, 99:1) and subsequently purified by distillation using a Kugelrohr apparatus (140 °C, 10 hPa, 1 h). The desired product was obtained as a colorless oil in 83% yield (152.1 mg).

Preparation of 0.1 M $MnCl_2$ solution

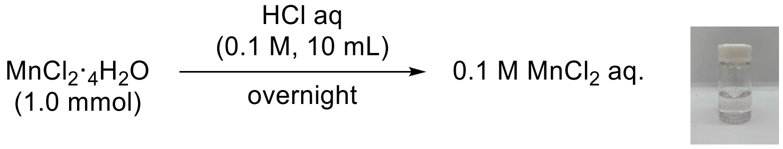

To a 20 mL vial bottle, $MnCl_2 \cdot 4H_2O$ (198 mg, 1 mmol) was dissolved in HCl aq (1 M, 10 mL). The solution was left under Ar in the dark overnight.

Preparation of 1.0 mM Mn NPs solution

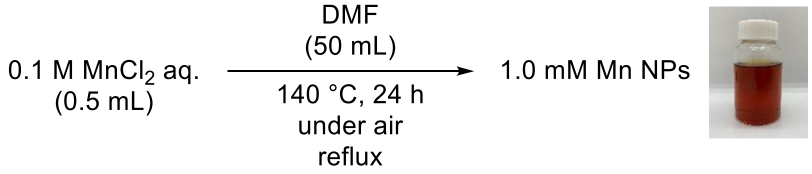

All the preparative operations were carried out under open air. To a 300-mL three-necked round bottom flask, *N,N*-dimethylformamide (DMF) (50 mL) preheated for 10 min was added, and the mixture was stirred at 1500 rpm and 140 °C. Then, 0.1 M $MnCl_2$ solution (500 μL) was added using micropipette, and the mixture was refluxed at 140 °C for 24 h. After reaction, the resulting solution was cooled to room temperature. The resulting solution was used as a 1.0 mM Mn NPs solution for catalytic reactions.

Preparation of samples for scanning tunnelling electron microscopy

For the before-reaction samples, DMF was removed from the Mn NP solution (1 mM, 1 mL) under vacuum, and the nanoparticles were dispersed in ethanol (2 mL). For the after-reaction samples, diglyme was removed by evaporation at 80°C and 30 hPa after the reaction. DMF (2

mL) was then added, and the reactants and products were extracted from the DMF layer using hexane (8 mL, five times). The hexane extracts were collected, and DMF was subsequently replaced with ethanol.

Preparation of samples for FT-IR, X-ray photoelectron spectroscopy, and X-ray diffraction

The DMF in the Mn NPs suspension was removed by evaporator at 80 °C and 30 hPa. The Mn NPs samples were prepared by placing the Mn NPs on a 5 mm × 5 mm Ag plate. For XRD measurements, the dried and solidified Mn NPs were sealed in an airtight cell within an Ar-filled bag.

Preparation of samples for XAS

DMF was removed from the Mn NPs with an evaporator, and the final liquid samples were prepared by adding DMF to obtain the required concentration of 40 mM. The solution (40 mM based on Mn content) was transferred to a polytetrafluoroethylene (PTFE) cell and the cell was filled with the reaction solution. Unless otherwise noted, XAS experiments were performed with the PTFE cell by transmitting X-rays through holes in the PTFE cell sealed with Kapton tapes (light path = 3 mm).

Preparation of samples for photoluminescence and UV-Vis spectroscopies

Samples (2 mL) of Mn NPs were prepared in DMF.

3. Results of Photoluminescence and UV-Vis spectroscopic analyses

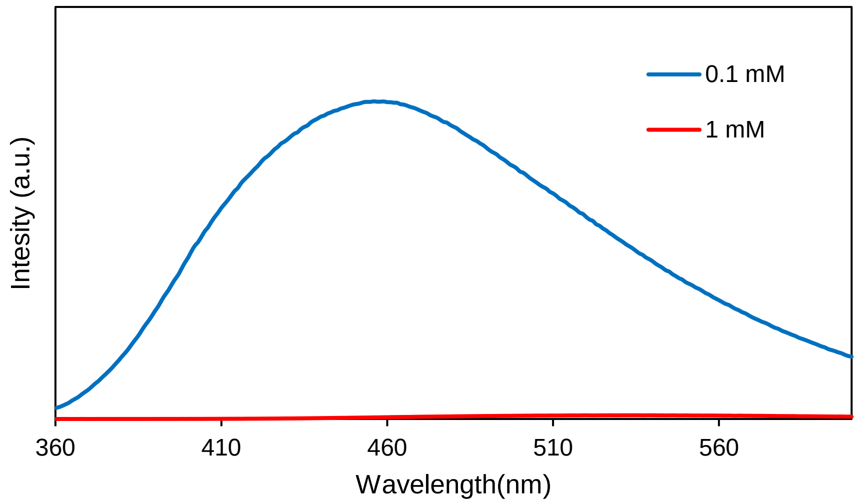

Fig. S1. Photoluminescence spectra of Mn NPs under irradiation with 350 nm excitation light (red line: 1 mM, blue line: 0.1 mM). The decrease in fluorescence intensity at 1 mM is caused by concentration quenching.

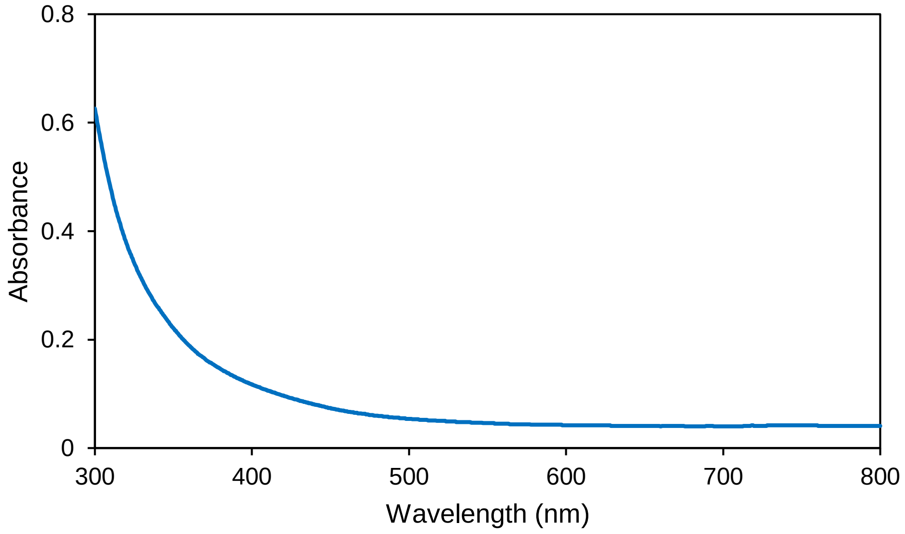

Fig. S2. UV-Vis absorption spectrum for Mn NPs (0.02 mM).

4. Results of XPS analysis

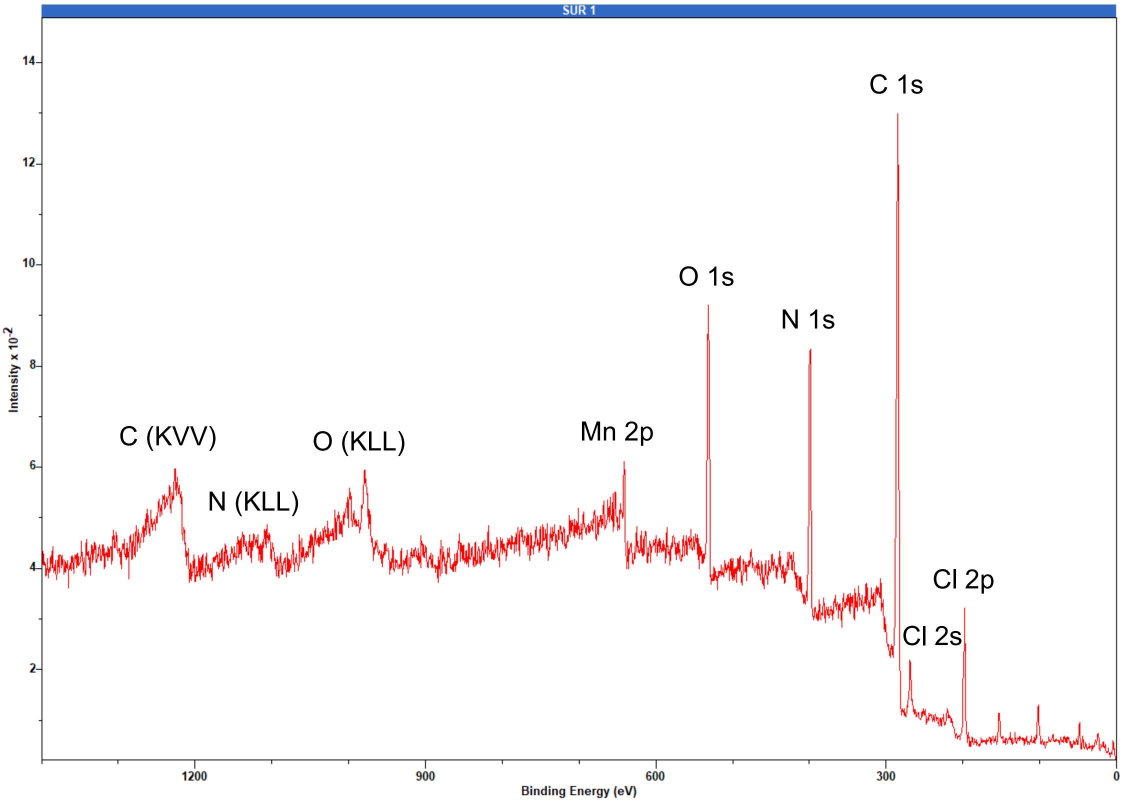

Fig. S3. Wide-scan XPS spectrum of Mn NPs before reaction.

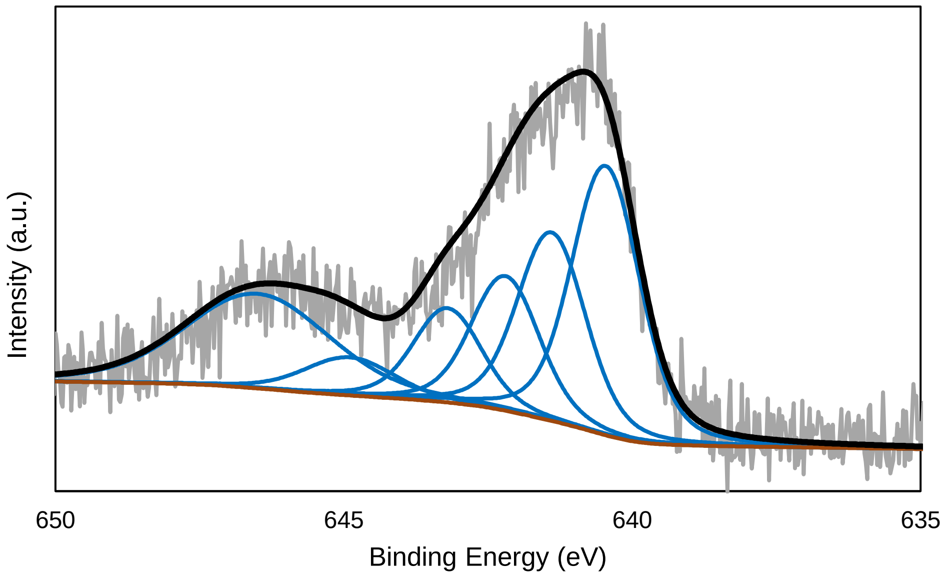

Fig. S4. XPS profile of the Mn $2p_{3/2}$ region for Mn NPs before reaction.

Table S1. Mn $2p_{3/2}$ spectral fitting parameters

Sample	Binding Energy (eV)	FWHM	Area (%)
Mn NPs Before reaction Mn $2p_{3/2}$	640.4	1.4	28.8
	641.4	1.4	20.1
	642.2	1.4	14.4
	643.2	1.4	10.1
	644.9	1.8	5.3
	646.5	3.0	21.3

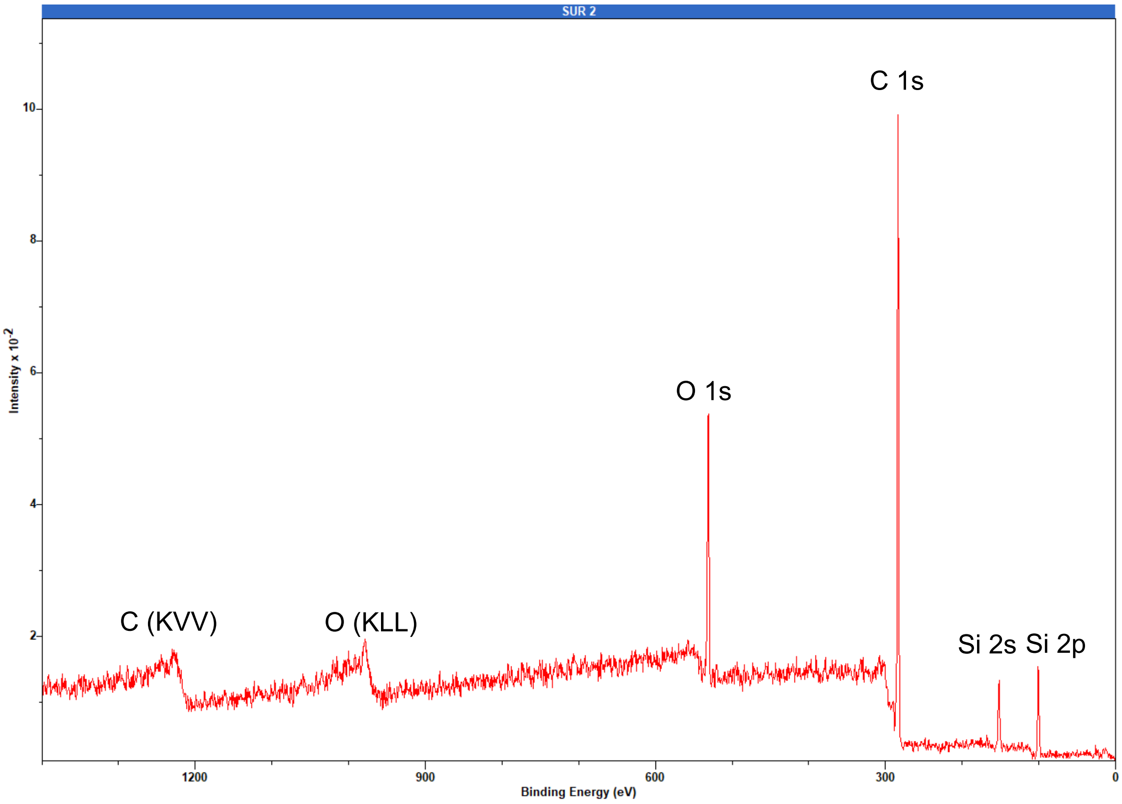

Fig. S5. Wide-scan XPS spectrum of Mn NPs after reaction. The Si compounds on the surface of the Mn NPs sample could not be completely removed, which indicates unanalysable spectra in the Mn 2p peak area.

5. Results of XAS analysis

Table S2. Curve-fitting analysis for Mn K-edge extended X-ray absorption fine structures (EXAFS) analysis of Mn NPs before and after reaction.

Sample	Shell	CN [a]	*R* (Å) [b]	σ^2 ($Å^2$) [c]	R_f [d]
Mn NPs before reaction	Mn-O	4.1	2.26	0.00111	0.016
Mn NPs after reaction	Mn-O	4.0	2.25	0.00070	0.022

[a]Coordination number. [b]Atomic distance. [c]Debye–Waller factor. [d]Residual factor.

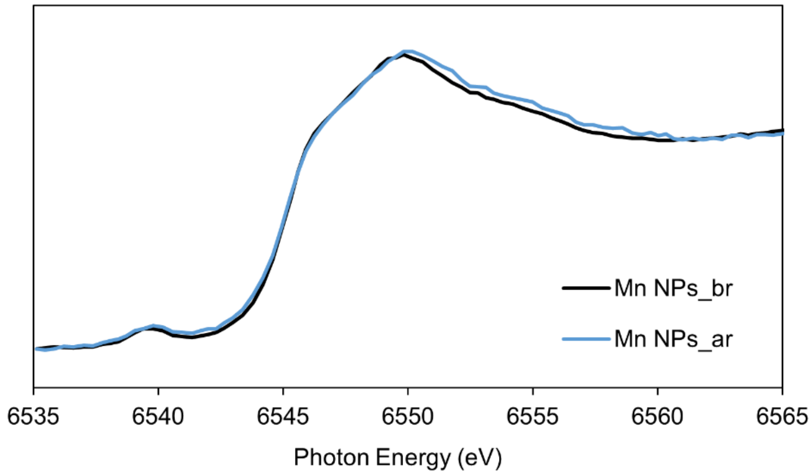

Fig. S6. Mn K-edge XAS of Mn NPs_before reaction (MnNPs_br) and Mn NPs_after reaction (MnNPs_ar). (E_0: MnNPs_br=6545.2 eV, MnNPs_ar=6545.2 eV).

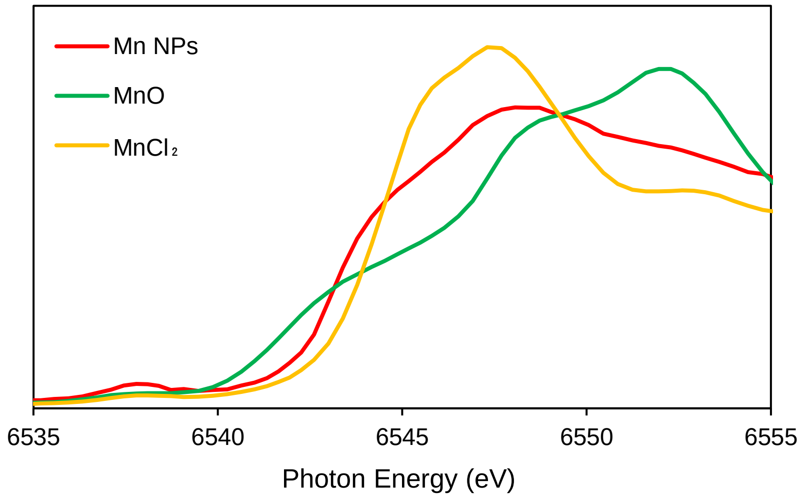

Fig. S7. Mn K-edge XANES of Mn NPs, MnO and $MnCl_2$

6. Recycling experiment procedure

Mn NPs (500 µL, 0.1 mol%) were added to a Schlenk tube and the DMF removed under vacuum. Then, 1-dodecene (**1a**: 0.5 mmol), methyldiphenylsilane (**2a**: 2.5 mmol), and diglyme (1 mL) as the solvent were added and the reaction was carried out at 130 °C under Ar for 24 h. The conversions and product yields were estimated using GC from the peak areas based on an internal standard. After performing the reaction, diglyme was removed by evaporation at 80 °C under 30 hPa. Next, hexane (8 mL) and DMF (2 mL) were added, and the products were recovered from the reaction mixture with hexane (8 mL, five times; upper layer, hexane; lower layer, DMF). The Mn NPs remained well dispersed in the DMF layer, which could be observed by photoluminescence under UV (352 nm) irradiation. The DMF layer was then evaporated (80 °C, 30 hPa) and only the catalyst was recovered. Recovered Mn NPs were added to a Schlenk tube and 1-dodecene (**1a**: 0.5 mmol), methyldiphenylsilane (**2a**: 2.5 mmol), and diglyme (1 mL) as solvent were added, and the next reaction was carried out at 130 °C under Ar for 24 h.

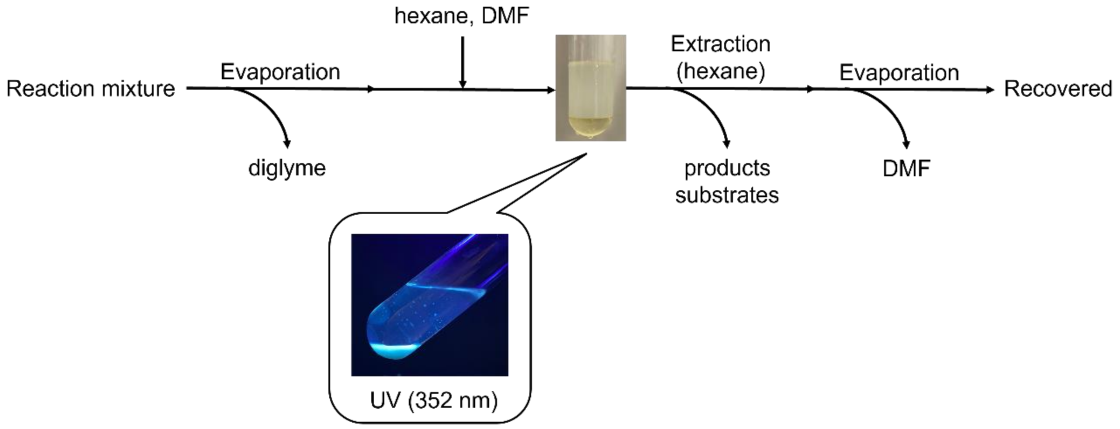

Scheme S1. Catalyst recycling procedure.

7. Other information

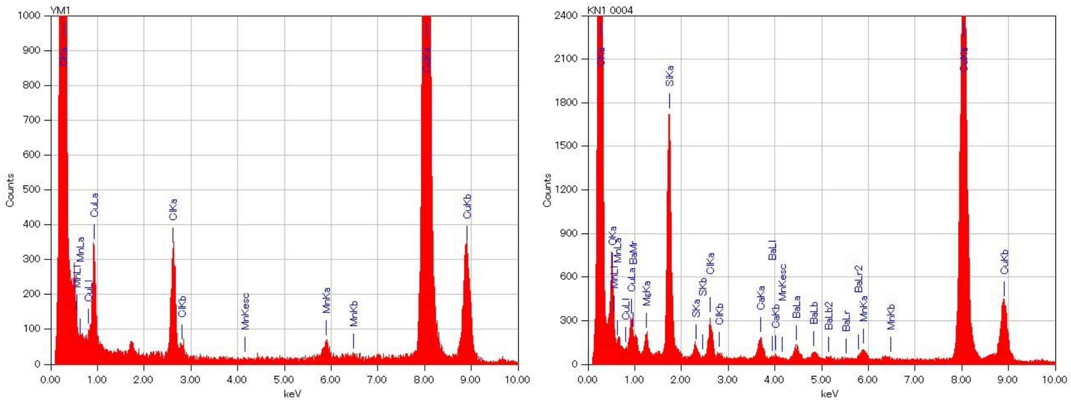

Fig. S8. Energy-dispersive X-ray spectroscopic analysis of Mn peaks (left: before reaction; right: after reaction).

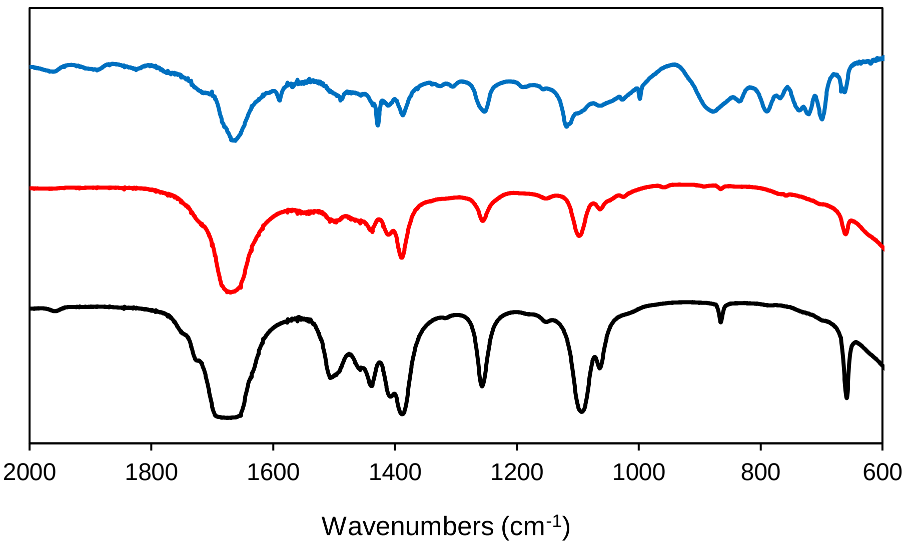

Fig. S9. FT-IR spectra of Mn NPs_ar (blue), Mn NPs_br (red), and DMF (black).

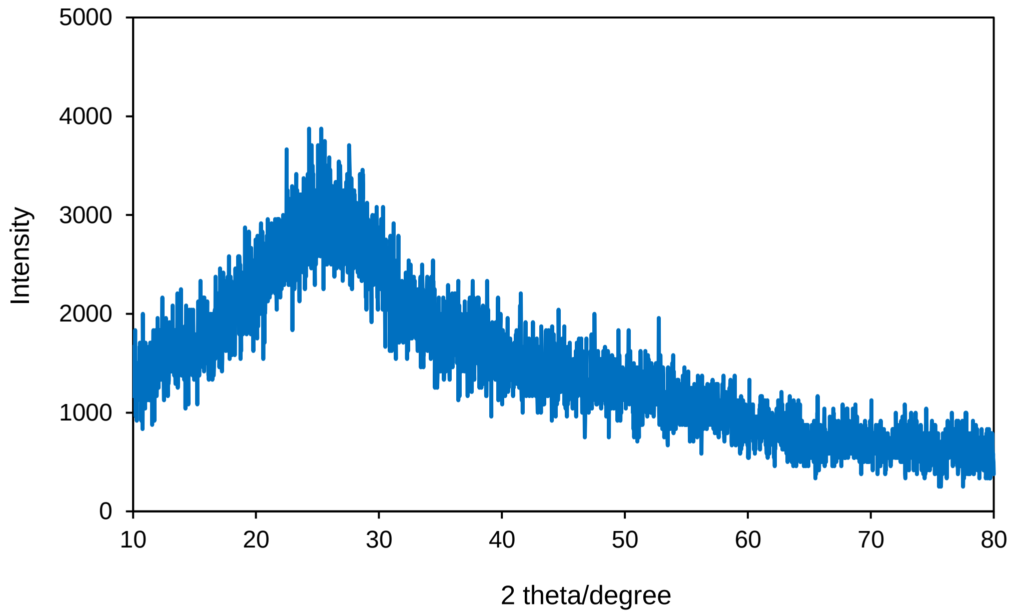

Fig. S10. XRD pattern for the Mn NPs before reaction.

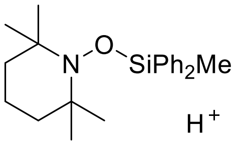

Chemical Formula: $C_{22}H_{32}NOSi^{+}$
Exact Mass: 354.2248

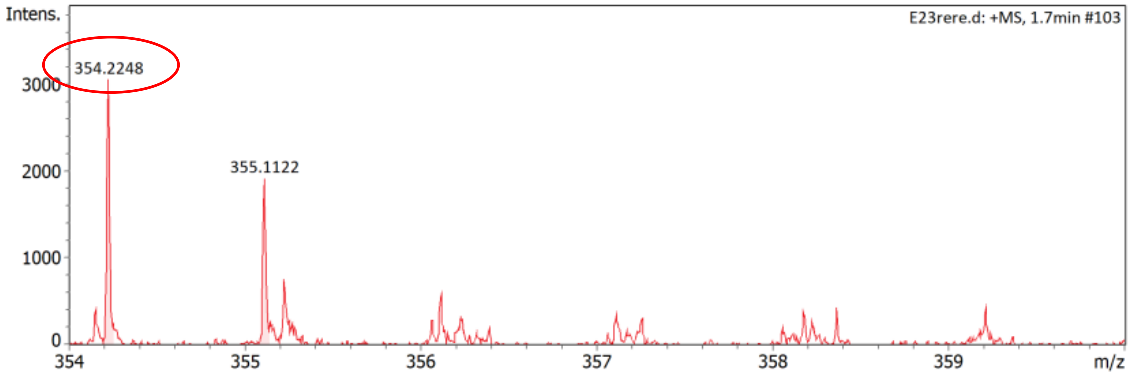

Fig. S11. The HRMS results for radical trapping with methyldiphenylsilane.
HRMS (ESI) *m/z* Calcd for $C_{22}H_{31}NOSi$ $[M+H]^{+}$ 354.2248, found 354.2248.

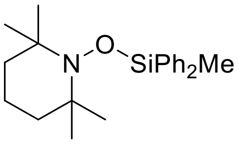

Chemical Formula: $C_{22}H_{31}NOSi$
m/z: 353 (100.0%), 354 (29.6%)

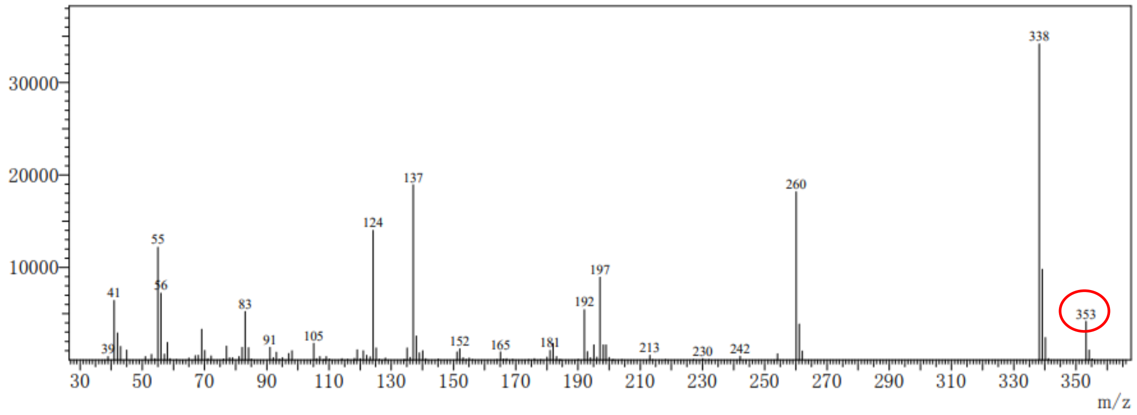

Fig. S12. The GC-MS results for radical trapping with methyldiphenylsilane.
GC-MS (EI) *m/z* (relative intensity) 353(12) $[M]^{+}$, 338(100), 137(55), 260(53).

Table S3. Manganese catalysts for alkene hydrosilylation.

Catalyst	Alkene	Silane	Temp. (°C)	Additive	Yield (%)	TON	Ref.
Mn NPs (0.005 mol %)	$C_{10}H_{21}$	$HSiPh_2Me$	130	None	79	15800	This work
**Cat 1** (0.5 mol %)	C_6H_{13}	$HSi(OEt)_3$	25	*t*-BuONa (1.5 mol %)	>95	190	11
**Cat 2** (0.5 mol %) **[Mn]** (1 mol %)	Myrcene	H_3SiPh	130	None	96	96	12
**Cat 3** (5 mol %)	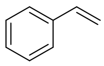	$HSiMe_2Bn$	60	None	95	19	13
**Cat 4** (3 mol %) **[Mn]** (6 mol %)	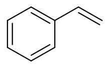	$HSiEt_3$	120	JackiePhos (6 mol %)	83	13.8	14
**Cat 4** (1 mol %) **[Mn]** (2 mol %)	C_6H_{13}	$HSiMe(OTMS)_2$	RT (UV)	None	85	42.5	15
**Cat 3** (2 mol %)	Ph	$HSiMe(OTMS)_2$	70	None	84	42	16
**Cat 4** (2.5 mol %) **[Mn]** (5 mol %)	C_6H_{13}	$HSi(OTMS)_3$	RT (White LEDs)	HFIP (5 mol %)	94	18.8	17

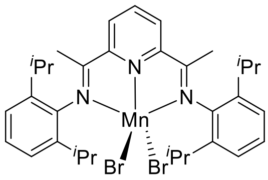

Cat 1

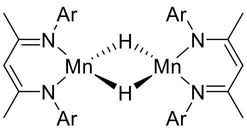

Cat 2

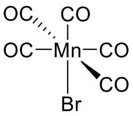

Cat 3

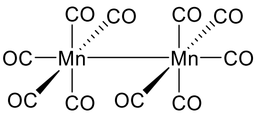

Cat 4

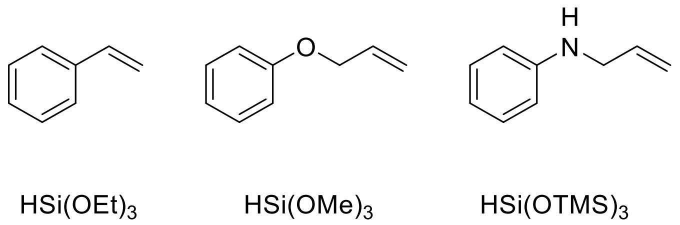

Fig. S13. Unsuccessful substrates.

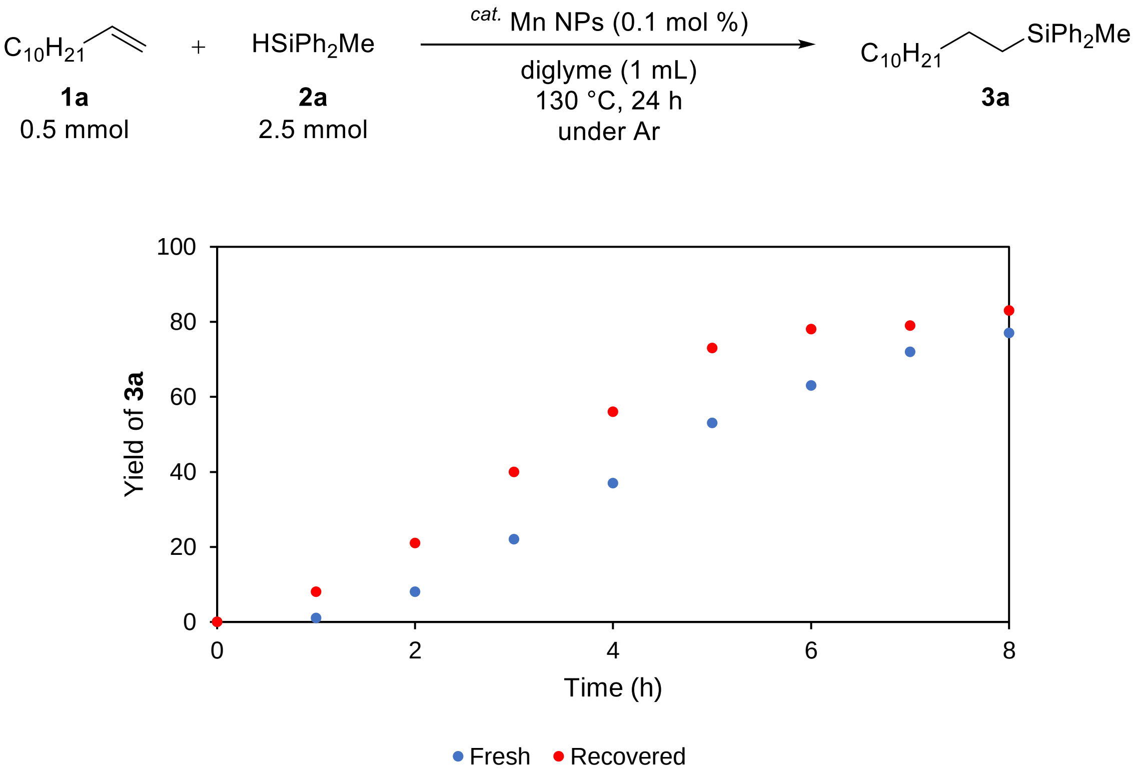

Fig. S14. Time course monitoring of hydrosilylation of 1-dodecene with methyldiphenylsilane.

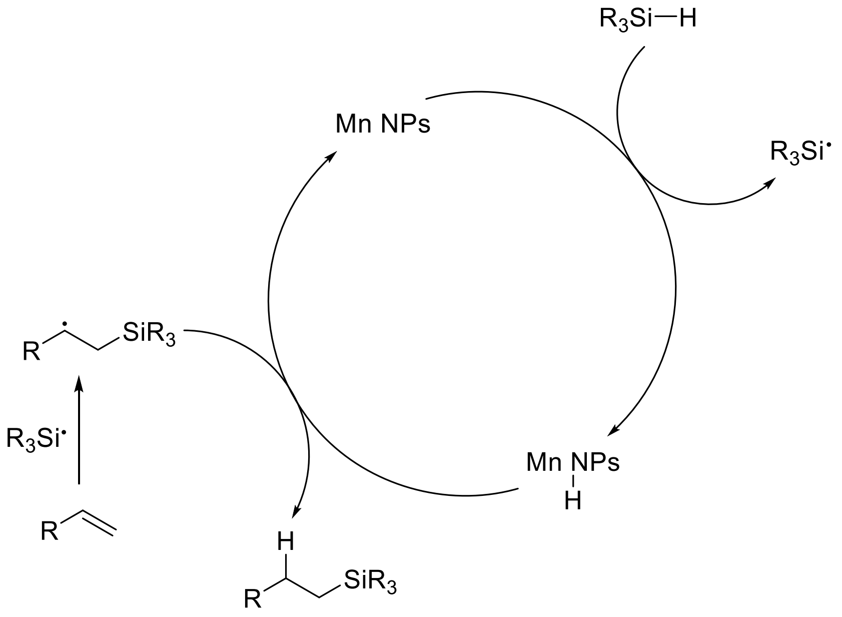

Fig. S15. A plausible reaction mechanism of Mn NPs-catalyzed alkene hydrosilylation.

8. Screening information

Table S4. Screening of silane ration

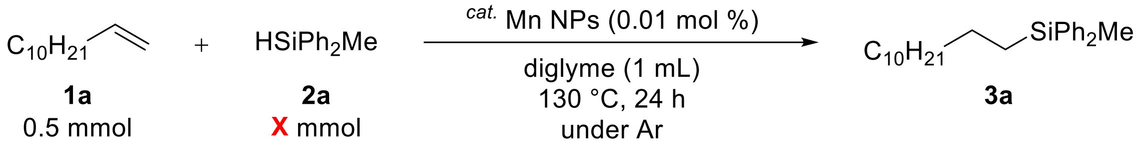

Entry	Silane amount	Conversion (%)		GC Yield (%)
		**1a**	**2a**	**3a**
1	0.5 mmol	>99	76	34
2	1.0 mmol	>99	50	55
3	1.5 mmol	97	34	59
4	2.0 mmol	94	34	62
5	2.5 mmol	>99	23	86

Reaction conditions: 1-dodecene (0.5 mmol) reacted with methyldiphenylsilane (X mmol) in the presence of Mn NPs (0.00005 mmol, 0.01 mol %) in diglyme (1 mL) at 130 °C for 24 h.

Table S5. Screening of catalyst amount

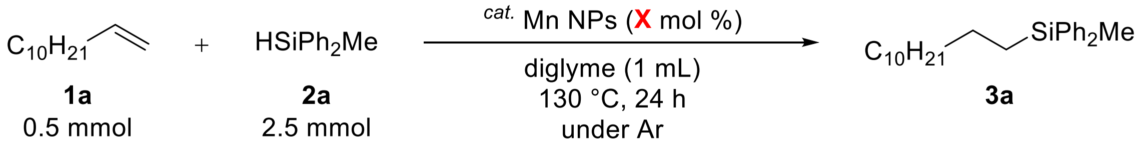

Entry	Catalyst amount	Conversion (%)		GC Yield (%)
		**1a**	**2a**	**3a**
1	0.01 mol %	>99	23	86
2	0.02 mol %	>99	44	79
3	0.05 mol %	>99	21	83
4	0.1 mol %	>99	25	79

Reaction conditions: 1-dodecene (0.5 mmol) reacted with methyldiphenylsilane (2.5 mmol) in the presence of Mn NPs (X mol %) in diglyme (1 mL) at 130 °C for 24 h.

Table S6. Effect of radical scavenger

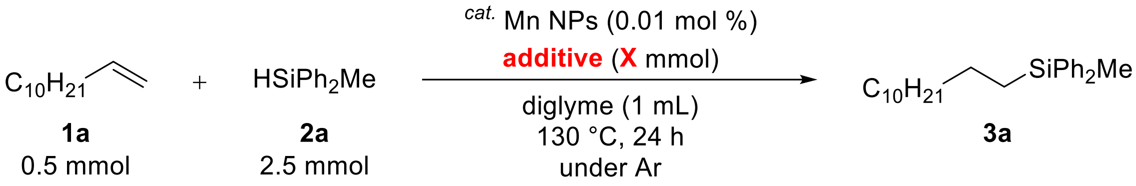

Entry	Additive	Conversion (%)		GC Yield (%)
		**1a**	**2a**	**3a**
1	none	>99	23	86
2	TEMPO (2.5 mmol)	24	>99	nd
3	TEMPO (0.2 mmol)	<1	23	nd
4	BHT (0.2 mmol)	<1	-	nd

Reaction conditions: 1-dodecene (0.5 mmol) reacted with methyldiphenylsilane (2.5 mmol) in the presence of Mn NPs (0.01 mol %) and **additive** (**X** mmol) in diglyme (1 mL) at 130 °C for 24 h; nd: not detected.

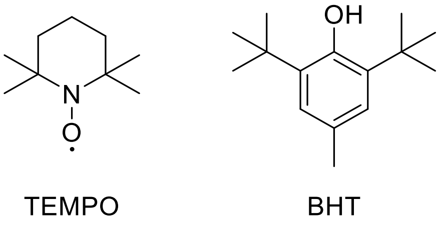

Table S7. Screening of catalyst species

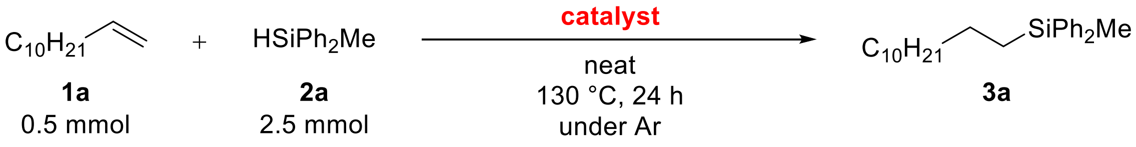

Entry	Catalyst	Conversion (%)		GC Yield (%)
		**1a**	**2a**	**3a**
1	Mn NPs (0.01 mol %)	96	26	70
2	$Mn_2(CO)_{10}$ (2.5 mol %)	92	-	59
3	HCl aq. (1 M, 50 μL, 10 mol %)	92	38	35
4	MnO (20 mol %)	<1	<1	nd
5	Mn NPs (0.01 mol %)[a]	-	-	32

Reaction conditions: 1-dodecene (0.5 mmol) reacted with methyldiphenylsilane (2.5 mmol) in the presence of **catalyst** (**X** mol %) at 130 °C for 24 h; nd: not detected. [a]Under air (balloon).

9. Characterization data

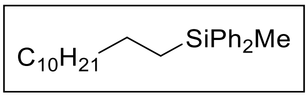

3a: The desired product was isolated by column chromatography (silica gel, hexane/ethyl acetate, 99:1) and subsequently purified by distillation using a Kugelrohr apparatus (140 °C, 10 hPa, 1 h). The desired product was obtained as a colorless oil in 83% yield (152.1 mg).

^{1}H-NMR (400 MHz, $CDCl_3$) δ: 7.53-7.49 (m, 4H), 7.39-7.32 (m, 6H), 1.39-1.16 (m, 20H), 1.05 (t, J = 8.0 Hz, 2H), 0.88 (t, J = 6.9 Hz, 3H), 0.53 (s, 3H); ^{13}C-NMR (100 MHz, CDCl3) δ: 137.5 (C), 134.4 (CH), 129.0 (CH), 127.7 (CH), 33.6 (CH_2), 31.9 (CH_2), 29.6 (CH_2), 29.6 (CH_2), 29.5 (CH_2), 29.3 (CH_2), 29.2 (CH_2), 23.7 (CH_2), 22.6 (CH_2), 14.1 (CH_2), 14.1 (CH_3), -4.4 (CH_3); IR (neat, cm^{-1}) 698, 730, 786, 1112, 1250, 1427, 1466, 2853, 2924; GC-MS (EI) *m/z* (relative intensity) 289(5) $[M-CH_3]^+$, 135(100), 121(25), 127(14); HRMS (EI) *m/z* calcd for $C_{25}H_{38}Si$ $[M-CH_3]^+$, 351.2508, found 351.2502.

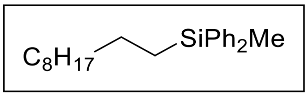

3b: The desired product was isolated by column chromatography (silica gel, hexane/ethyl acetate, 99:1) and subsequently purified by distillation using a Kugelrohr apparatus (130 °C, 0.3 mmHg, 1 h). The desired product was obtained as a colorless oil in 77% yield (130.3 mg).

^{1}H-NMR (400 MHz, $CDCl_3$) δ: 7.53-7.51 (m, 4H), 7.39-7.32 (m, 6H), 1.34-1.24 (m, 16H), 1.07-1.05 (m, 2H), 0.88 (t, J = 6.9 Hz, 3H), 0.54 (s, 3H);^{13}C-NMR (100 MHz, $CDCl_3$) δ: 137.5 (C), 134.4(CH), 129.0 (CH), 127.7 (CH), 33.6 (CH_2), 31.8 (CH_2), 29.6 (CH_2), 29.5 (CH_2), 29.3 (CH_2), 29.2 (CH_2), 23.7 CH_2), 22.6 (CH_2), 14.1 (CH_3, CH_2), -4.4 (CH_3); GC-MS (EI) *m/z* (relative intensity) 323(1) $[M-CH_3]^+$, 197(100), 198(20), 183(9).

The spectroscopic data were consistent with the reported values.[1]

Miura, H.; Doi, M.; Yasui, Y.; Masaki, Y.; Nishio, H.; Shishido, T. Diverse Alkyl-Silyl Cross-Coupling via Homolysis of Unactivated C(sp^3)-O Bonds with the Cooperation of Gold Nanoparticles and Amphoteric Zirconium Oxides. *J. Am. Chem. Soc.* **2023**, *145*, 4613–4625.

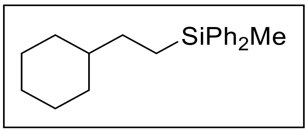

3c: The desired product was isolated by column chromatography on silica gel using hexane as the eluent, followed by purification via distillation using a Kugelrohr apparatus (140 °C, 10 hPa, 1 h). The desired product was obtained as a colorless oil in 68% yield (104.9 mg).

^{1}H-NMR (400 MHz, $CDCl_3$) δ: 7.52-7.49 (m, 4H), 7.38-7.31 (m, 6H), 1.71-1.64 (m, 5H), 1.28-1.03 (m, 8H), 0.86-0.82 (m, 2H), 0.53 (s, 3H); ^{13}C-NMR (100 MHz, $CDCl_3$) δ: 137.5 (C), 134.4 (CH), 129.0 (CH), 127.7 (CH), 40.7 (CH), 32.9 (CH_2), 31.1 (CH_2), 26.7 (CH_2), 26.4 (CH_2), 11.0 (CH_2), -4.5 (CH_3); IR (neat, cm^{-1}) 698, 731, 788, 1112, 1250, 1427, 1449, 2849, 2920; HRMS (ESI) *m/z* Calcd for $C_{21}H_{29}Si$ $[M+H]^+$ 309.2033, found 309.2032.

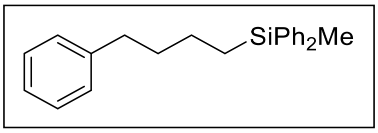

3d: The desired product was purified by distillation using a Kugelrohr apparatus (130°C, 0.3 mmHg, 1 h), and subsequently isolated by column chromatography on silica gel using hexane as the eluent. The desired product was obtained as a colorless oil in 65% yield (107.4 mg).
^{1}H-NMR (400MHz; $CDCl_3$) δ: 7.51-7.49 (m, 4H), 7.38-7.31 (m, 6H), 7.25-7.23 (m, 2H), 7.17-7.11 (m, 3H), 2.57 (t, J = 7.8 Hz, 2H), 1.69-1.62 (m, 2H), 1.46-1.42 (m, 2H), 1.12-1.08 (m, 2H), 0.53 (s, 3H); ^{13}C-NMR (100MHz; $CDCl_3$) δ: 142.6 (C), 137.3 (C), 134.4 (CH), 129.0 (CH), 128.3 (CH), 128.2 (CH), 127.7 (CH), 125.5 (CH), 35.4 (CH_2), 35.3 (CH_2), 23.5 (CH_2), 13.9 (CH_2), -4.4 (CH_3); GC-MS (EI) *m/z* (relative intensity) 315 (0.2) $[M-CH_3]^+$ 197 (100) 252 (36) 198 (19).
The spectroscopic data were consistent with the reported values.[2]

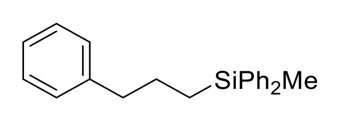

3e: The desired product was isolated by column chromatography (silica gel, hexane/ethyl acetate, 99:1). The desired product was obtained as a colorless oil in 66% yield (104.4 mg).

^{1}H-NMR (400 MHz, $CDCl_3$) δ: 7.50-7.46 (4H, m), 7.37-7.32 (6H, m), 7.26-7.24 (2H, m), 7.17-7.12 (3H, m), 2.64 (2H, t, J = 7.6 Hz), 1.72-1.68 (2H, m), 1.12-1.08 (2H, m), 0.53 (3H, s); ^{13}C-NMR (100 MHz, $CDCl_3$) δ: 142.3 (C), 137.1 (C), 134.4 (CH), 129.0 (CH), 128.5 (CH), 128.2 (CH), 127.7 (CH), 125.6 (CH), 39.7 (CH_2), 25.8 (CH_2), 13.8 (CH_2), -4.4 (CH_3); IR (neat, cm^{-1}) 698, 731, 789, 1112, 1251, 1427, 1453, 2858, 2926, 3024, 3068; GC-MS (EI) *m/z* (relative intensity) 289(5) $[M-CH_3]^+$, 135(100), 121(25), 127(14); HRMS (ESI) *m/z* Calcd for $C_{22}H_{24}SiNa$ $[M+Na]^+$ 339.1539, found 339.1540.

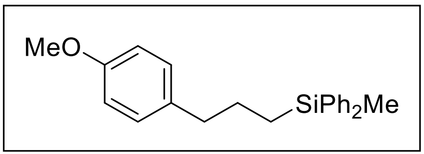

3f: The desired product was isolated by column chromatography (silica gel, hexane/ethyl acetate, 99:1). The desired product was obtained as a colorless oil in 77% yield (133.4 mg).

^{1}H-NMR (400 MHz, $CDCl_3$) δ: 7.52-7.46 (4H, m), 7.38-7.30 (6H, m), 7.03 (2H, d, J = 8.7 Hz), 6.80 (2H, d, J = 8.7 Hz), 3.77 (3H, s), 2.58 (2H, t, J = 7.5 Hz), 1.67-1.65 (2H, m), 1.10-1.07 (2H, m), 0.53 (3H, s); ^{13}C-NMR (100 MHz, $CDCl_3$) δ: 157.6 (C), 137.2 (C), 134.4 (CH), 129.3 (CH), 129.0 (CH), 127.8 (C), 127.7 (CH), 113.5 (CH), 55.2 (CH_3), 38.7 (CH_2), 25.9 (CH_2), 13.7 (CH_2), -4.4 (CH_3); IR (neat, cm^{-1}) 700, 730, 788, 1037, 1111, 1248, 1427, 1512, 2927, 3068; HRMS (ESI) *m/z* Calcd for $C_{23}H_{26}OSiNa$ $[M+Na]^+$ 369.1645, found 369.1652.

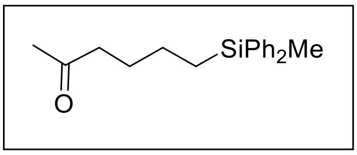

3g: The desired product was isolated by column chromatography (silica gel, hexane/ethyl acetate, 8:2). The desired product was obtained as a colorless oil in 50% yield (74.1 mg).

^{1}H-NMR (400 MHz, $CDCl_3$) δ: 7.51-7.49 (4H, m), 7.38-7.33 (6H, m), 2.40 (2H, t, J = 7.4 Hz), 2.10 (3H, s), 1.66-1.59 (2H, m), 1.42-1.34 (2H, m), 1.07 (2H, t, J = 8.5 Hz), 0.54 (3H, s); ^{13}C-NMR (100 MHz, $CDCl_3$) δ: 209.2 (C), 137.1 (C), 134.3 (CH), 129.1 (CH), 127.8 (CH), 43.4 (CH_2), 29.8 (CH_3), 27.6 (CH_2), 23.4 (CH_2), 14.0 (CH_2), -4.8 (CH_3); GC-MS (EI) *m/z* (relative intensity) 239(2) $[M]^+$ -C_3H_5O, 197(100), 219(56), 137(28).

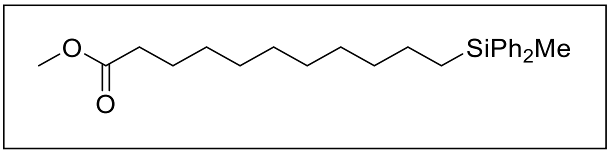

3h: The desired product was isolated by column chromatography (silica gel, hexane/ethyl acetate, 99:1) and subsequently purified by distillation using a Kugelrohr apparatus (200 °C, 10 hPa, 0.5 h). The desired product was obtained as a colorless oil in 59% yield (117.0 mg).

^{1}H-NMR (400 MHz, $CDCl_3$) δ: 7.52-7.49 (4H, m), 7.37-7.32 (6H, m), 3.66 (3H, s), 2.29 (2H, t, J = 7.5 Hz), 1.60 (2H, t, J = 7.3 Hz), 1.34-1.26 (14H, m), 1.06-1.04 (2H, m), 0.53 (3H, s). ^{13}C-NMR (100 MHz, $CDCl_3$) δ: 174.3 (C), 137.4 (C), 134.4 (CH), 129.0 (CH), 127.7 (CH), 51.4 (CH_3), 34.0 (CH_2), 33.6 (CH_2), 29.4 (CH_2), 29.4 (CH_2), 29.2 (CH_2), 29.1 (CH_2), 29.1 (CH_2), 24.9 (CH_2), 23.7 (CH_2), 14.1 (CH_2), -4.4 (CH_3); IR (neat, cm^{-1}) 699, 787, 1112, 1250, 1427, 1740, 2851, 2922; HRMS (ESI) *m/z* Calcd for $C_{25}H_{36}O_2SiNa$ $[M+Na]^+$ 419.2377, found 419.2379.

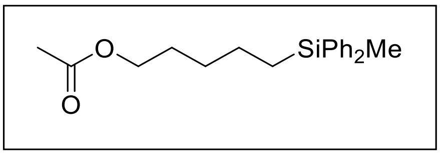

3i: The desired product was isolated by column chromatography (silica gel, hexane/ethyl acetate, 95:5). The desired product was obtained as a colorless oil in 60% yield (97.9 mg).

^{1}H-NMR (400 MHz, $CDCl_3$,) δ: 7.52-7.31 (m, 10H), 4.01 (t, J = 6.7 Hz, 2H), 2.01 (s, 3H), 1.60 (t, J = 6.9 Hz, 2H), 1.40-1.39 (m, 4H), 1.09-1.01 (m, 2H), 0.53 (s, 3H); ^{13}C-NMR (100 MHz, $CDCl_3$) δ: 171.1 (C), 137.2 (C), 134.3 (CH), 129.0 (CH), 127.7 (CH), 64.5 (CH_2), 29.8 (CH_2), 28.1 (CH_2), 23.5 (CH_2), 20.9 (CH_3), 14.1 (CH_2), -4.5 (CH_3); GC-MS (EI) *m/z* (relative intensity) 311 (1) [M-CH_3] $^{+}$ 197 (100) 179 (33) 249 (22); HRMS (ESI) *m/z* Calcd for $C_{20}H_{26}O_2SiNa$ $[M+Na]^+$ 350.1621, found 350.1629.

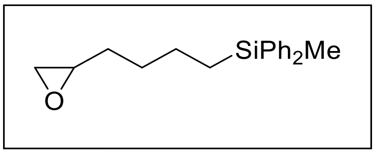

3j: The desired product was isolated by column chromatography (silica gel, hexane/ethyl acetate, 9:1). The desired product was obtained as a colorless oil in 73% yield (108.2 mg).

^{1}H-NMR (400 MHz, $CDCl_3$) δ: 7.52-7.51 (m, 4H), 7.40-7.33 (m, 6H), 2.87-2.85 (m, 1H), 2.71 (dd, J = 5.0, 4.0 Hz, 1H), 2.42 (dd, J = 5.0, 2.7 Hz, 1H), 1.58-1.41 (m, 6H), 1.11-1.07 (m, 2H), 0.55 (s, 3H) ; ^{13}C-NMR (100 MHz, $CDCl_3$) δ: 137.2 (C), 134.4 (CH), 129.1 (CH), 127.7 (CH), 52.2 (CH), 47.0 (CH_2), 32.0 (CH_2), 29.7 (CH_2), 23.6 (CH_2), 14.1 (CH_2), -4.5 (CH_3); IR (neat, cm^{-1}) 699, 732, 787, 1112, 1251, 1427, 2924; HRMS (ESI) *m/z* Calcd for $C_{19}H_{24}OSiNa$ $[M+Na]^+$ 319.1489, found 319.1474.

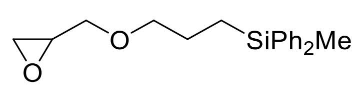

3k: The desired product was isolated by column chromatography (silica gel, hexane/ethyl acetate, 9:1). The desired product was obtained as a colorless oil in 77% yield (120.3 mg).

^{1}H-NMR (400 MHz, $CDCl_3$) δ: 7.52-7.50 (4H, m), 7.39-7.32 (6H, m), 3.66 (1H, dd, *J* = 11.5, 3.1 Hz), 3.48-3.43 (2H, m), 3.33 (1H, dd, *J* = 11.5, 5.9 Hz), 3.14-3.10 (1H, m), 2.78 (1H, dd, *J* = 5.0, 4.1 Hz), 2.58 (1H, dd, *J* = 5.0, 2.8 Hz), 1.71-1.63 (2H, m), 1.10-1.06 (2H, m), 0.55 (3H, s); ^{13}C-NMR (100 MHz, $CDCl_3$) δ: 136.9 (C), 134.4 (CH), 129.1 (CH), 127.8 (CH), 74.1 (CH_2), 71.3 (CH_2), 50.8 (CH), 44.3 (CH_2), 24.0 (CH_2), 10.1 (CH_2), -4.5 (CH_3); IR (neat, cm^{-1}) 700, 732, 786, 1112, 1251, 1427, 2869, 2932; HRMS (ESI) *m/z* calcd for $C_{19}H_{24}O_2SiNa$ $[M+Na]^+$, 335.1438, found 335.1445.

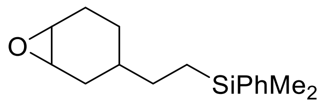

3l: The desired product was isolated by column chromatography (silica gel, hexane/ethyl acetate, 95:5). The desired product was obtained as a colorless oil in 64% yield (83.3 mg).

^{1}H-NMR (400 MHz,$CDCl_3$) δ: 7.50-7.48 (m, 2H), 7.35-7.34 (m, 3H), 3.16-3.10 (m, 2H), 2.17-2.13 (m, 1H), 2.07-1.93 (m, 1H), 1.83-1.60 (m, 1H), 1.52-0.79 (m, 6H), 0.73-0.67 (m, 2H), 0.24 (s, 6H); ^{13}C-NMR (100 MHz, $CDCl_3$) δ: 139.3 (C), 133.4 (CH), 128.7 (CH), 127.6 (CH), 53.2 (CH), 52.7 (CH), 51.9 (CH), 51.9 (CH), 35.4 (CH), 32.4 (CH), 31.5 (CH_2), 30.6 (CH_2), 30.3 (CH_2), 30.1 (CH_2), 26.6 (CH_2), 25.3 (CH_2), 23.9 (CH_2), 23.5 (CH_2), 12.5 (CH_2), 12.4 (CH_2), -3.1 (CH_3), -3.17 (CH_3), -3.21 (CH_3); GC-MS (EI) *m/z* (relative intensity) 245 (1) $[M-CH_3]^+$ 135 (100) 137 (34) 136 (14).

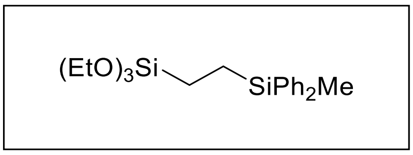

3m: The desired product was isolated by distillation using a Kugelrohr apparatus (160 °C, 10 hPa, 1 h). The desired product was obtained as a colorless oil in 73% yield (141.8 mg).

^{1}H-NMR (400 MHz, $CDCl_3$) δ: 7.51-7.50 (m, 2H), 7.34-7.34 (m, 3H), 3.80-3.78 (m, 6H), 1.24-1.18 (m, 9H), 0.79-0.76 (m, 2H), 0.56-0.54 (m, 2H), 0.26 (s, 6H); ^{13}C-NMR (100 MHz, $CDCl_3$) δ: 139.0 (C), 133.6 (CH), 128.7 (CH), 127.6 (CH), 58.3 (CH_2), 18.2 (CH_3), 6.7 (CH_2), 2.4 (CH_2), -3.7 (CH_3); GC-MS (EI) *m/z* (relative intensity) 311 (45) $[M-CH_3]^+$ 135 (100) 205 (53) 311 (45).

The spectroscopic data were consistent with the reported values.[5]

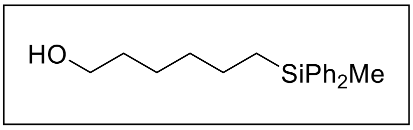

3n: The desired product was isolated by column chromatography (silica gel, hexane/ethyl acetate, 8:2). The desired product was obtained as a colorless oil in 69% yield (102.9 mg).

^{1}H-NMR (400 MHz, $CDCl_3$) δ: 7.52-7.50 (4H, m), 7.39-7.32 (6H, m), 3.61 (2H, t, J = 6.6 Hz), 1.53-1.51 (2H, m), 1.38-1.34 (6H, m), 1.21 (1H, br), 1.08-1.06 (2H, m), 0.54 (3H, s); ^{13}C-NMR (100 MHz, $CDCl_3$) δ: 137.3 (C), 134.4 (CH), 129.0 (CH), 127.7 (CH), 62.9 (CH_2), 33.2 (CH_2), 32.6 (CH_2), 25.2 (CH_2), 23.7 (CH_2), 14.0 (CH_2), -4.5 (CH_3); IR (neat, cm-^{1}) 699, 731, 787, 1112, 1250, 1427, 2926, 3333; HRMS (ESI) *m/z* Calcd for $C_{19}H_{26}OSiNa$ $[M+Na]^+$ 321.1645, found 321.1642.

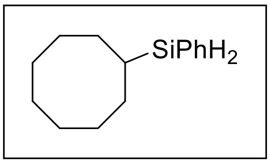

3o: The desired product was isolated by column chromatography (silica gel, hexane 100%). The desired product was obtained as a colorless oil in 33% yield (36.0 mg).

^{1}H-NMR (400 MHz, $CDCl_3$) δ: 7.58-7.56 (2H, m), 7.37-7.34 (3H, m), 4.19 (2H, d, J = 3.3 Hz), 1.84-1.43 (14H, m), 1.29-1.25 (1H, m); ^{13}C-NMR (100 MHz, $CDCl_3$) δ: 135.5 (CH), 132.6 (C), 129.4 (CH), 127.8 (CH), 28.6 (CH_2), 27.3 (CH_2), 26.8 (CH_2), 26.5 (CH_2),20.1 (CH); GC-MS (EI) *m/z* (relative intensity) 218 (0.8) $[M]^+$ 140 (100) 107 (92) 112 (60).

The spectroscopic data were consistent with the reported values.[6]

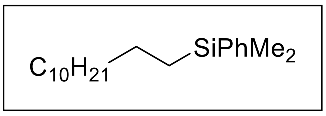

3p: The desired product was isolated by column chromatography (silica gel, hexane 100%) and subsequently purified by distillation using a Kugelrohr apparatus (150 °C, 0.3 mmHg, 30 min). The desired product was obtained as a colorless oil in 63% yield (95.9 mg).

^{1}H-NMR (400 MHz ; $CDCl_3$) δ: 7.52-7.33 (m, 5H), 1.30-1.26 (m, 20H), 0.88 (t, J = 6.9 Hz, 3H), 0.73 (t, J = 7.8 Hz, 2H), 0.25 (s, 6H); ^{13}C-NMR (100 MHz ; $CDCl_3$) δ: 139.7 (C), 133.5 (CH), 128.6 (CH), 127.6 (CH), 33.6 (CH_2), 31.9 (CH_2), 31.7 (CH_2), 29.6 (CH_2), 29.6 (CH_2), 29.5 (CH_2), 29.3 (CH_2), 29.3 (CH_2), 23.8 (CH_2), 22.6 (CH_2), 15.6 (CH_2), 14.1 (CH_3), -3.0 (CH_3); GC-MS (EI) *m/z* (relative intensity) 289 (4) $[M\text{-}CH_3]^+$ 135 (100) 121 (26) 127 (15).

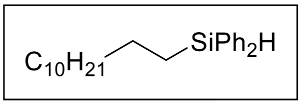

3q: The desired product was isolated by column chromatography (silica gel, hexane 100%) and subsequently purified by distillation using a Kugelrohr apparatus (110 °C, 0.3 mmHg, 30 min). The desired product was obtained as a colorless oil in 82% yield (144.5 mg).

^{1}H-NMR (400 MHz, $CDCl_3$) δ: 7.56-7.54 (m, 4H), 7.38-7.35 (m, 6H), 4.84 (t, *J* = 3.7 Hz, 1H), 1.47-1.11 (m, 22H), 0.88 (t, *J* = 6.9 Hz, 3H); ^{13}C-NMR (100 MHz, $CDCl_3$) δ: 135.1 (CH), 134.7 (C), 129.4 (CH), 127.9 (CH), 33.1 (CH_2), 31.9 (CH_2), 29.6 (CH_2), 29.6 (CH_2), 29.5 (CH_2), 29.3 (CH_2), 29.2 (CH_2), 24.3 (CH_2), 22.6 (CH_2), 14.1 (CH_3), 12.1 (CH_2); GC-MS (EI) *m/z* (relative intensity) 275 (1) $[M-C_6H_5]^+$ 183 (100) 175 (32) 105 (19).

The spectroscopic data were consistent with the reported values.[7]

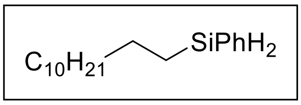

3r: The desired product was isolated by column chromatography (silica gel, hexane 100%). The desired product was obtained as a colorless oil in 82% yield (113.3 mg).

^{1}H-NMR (400 MHz, $CDCl_3$) δ: 7.58-7.55 (m, 2H), 7.38-7.34 (m, 3H), 4.28 (t, *J* = 3.7 Hz, 2H), 1.47-1.43 (m, 2H), 1.36-1.25 (m, 18H), 0.95-0.92 (m, 2H), 0.88 (t, *J* = 6.9 Hz, 3H); ^{13}C-NMR (100 MHz, $CDCl_3$) δ: 135.1 (CH), 132.8 (C), 129.4 (CH), 127.9 (CH), 32.8 (CH_2), 31.9 (CH_2), 29.6 (CH_2), 29.5 (CH_2), 29.3 (CH_2), 29.2 (CH_2), 25.0 (CH_2), 22.6 (CH_2), 14.2 (CH_3), 9.9 (CH_2); GC-MS (EI) *m/z* (relative intensity) 275 (0.03) $[M-H]^+$ 99 (100) 107 (54) 113 (38).

The spectroscopic data were consistent with the reported values.[8]

10. NMR-Spectra

3a ^{1}H-NMR

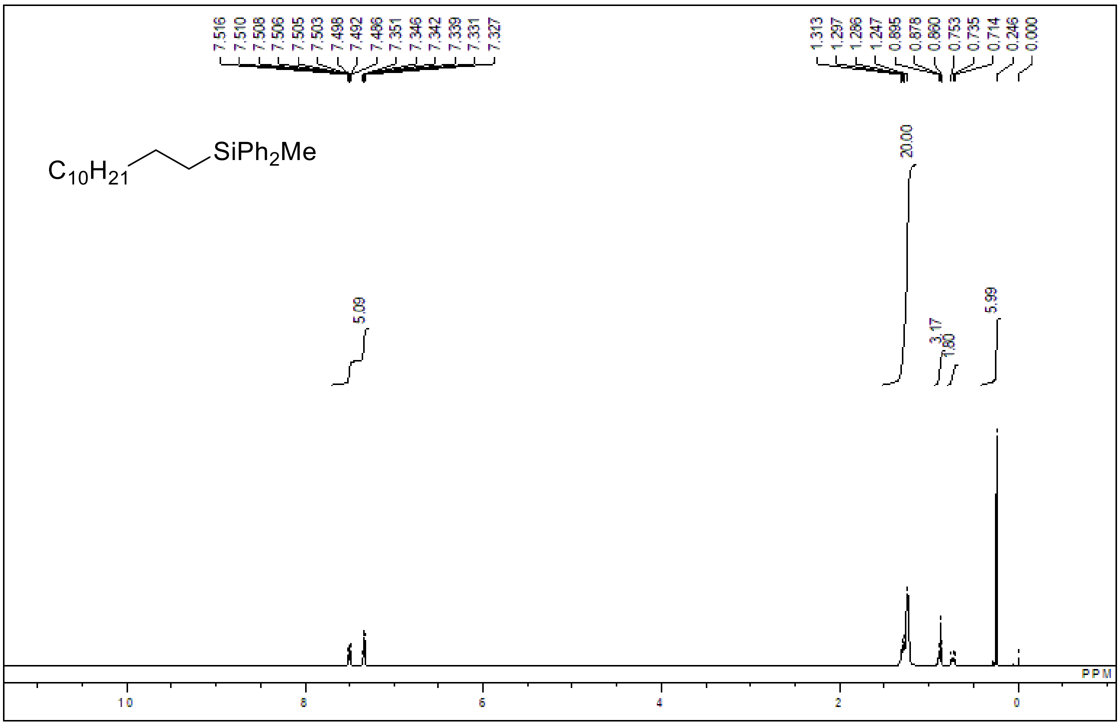

^{13}C-NMR

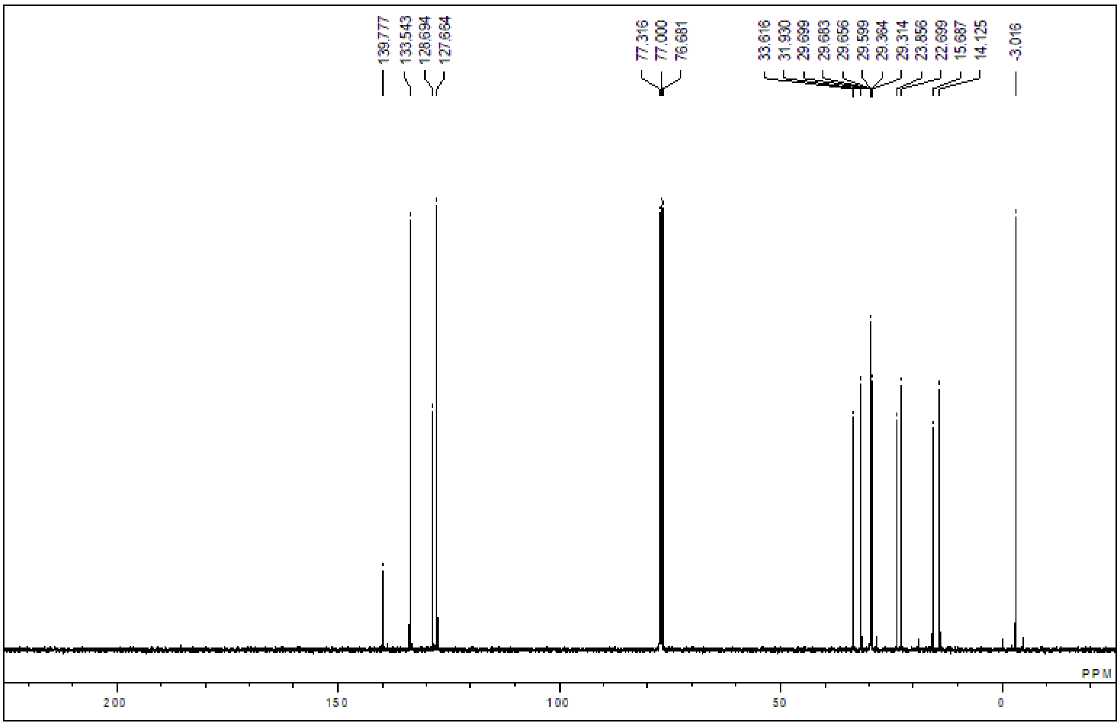

3b ^{1}H-NMR

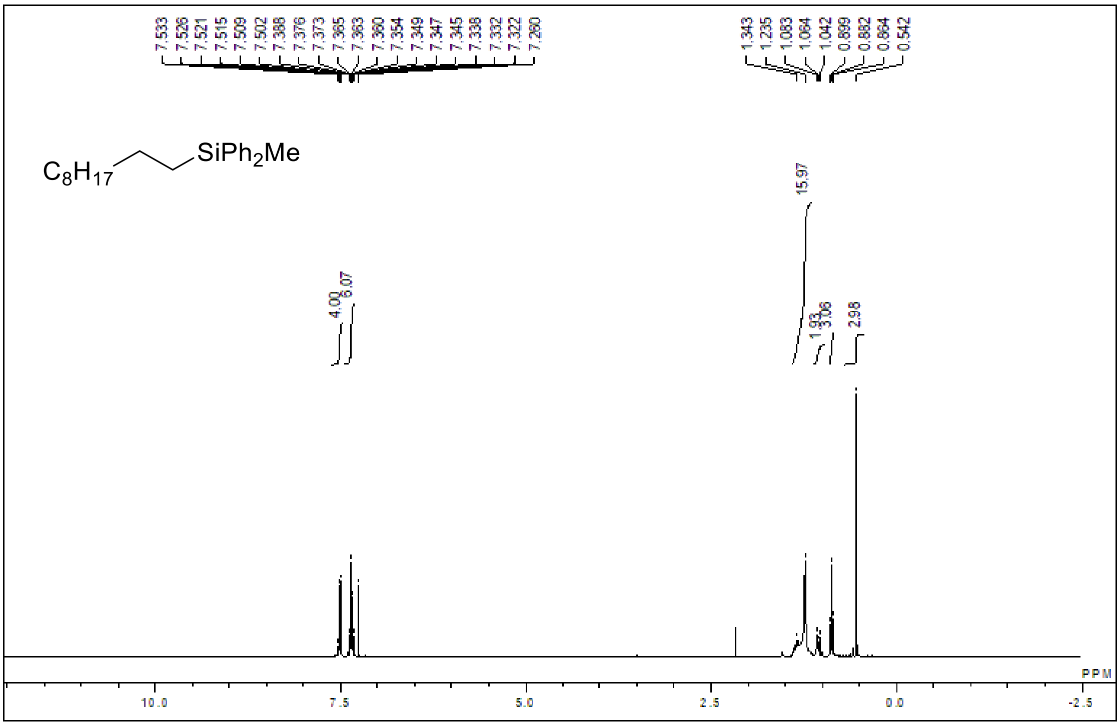

^{13}C-NMR

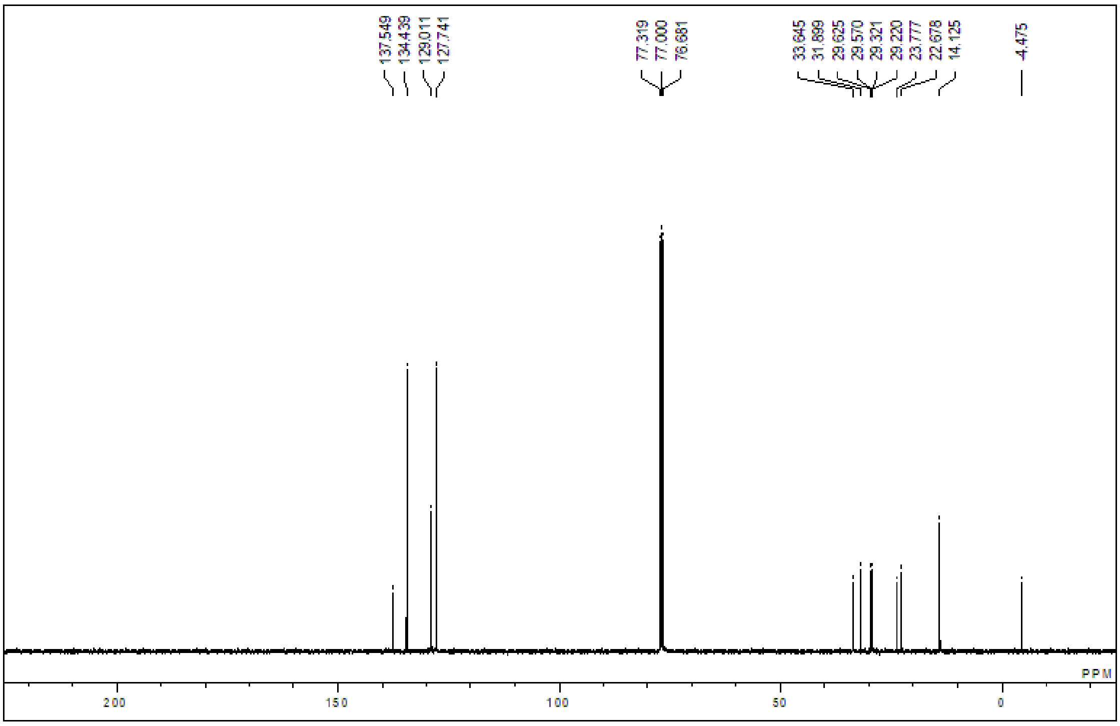

3c ^{1}H-NMR

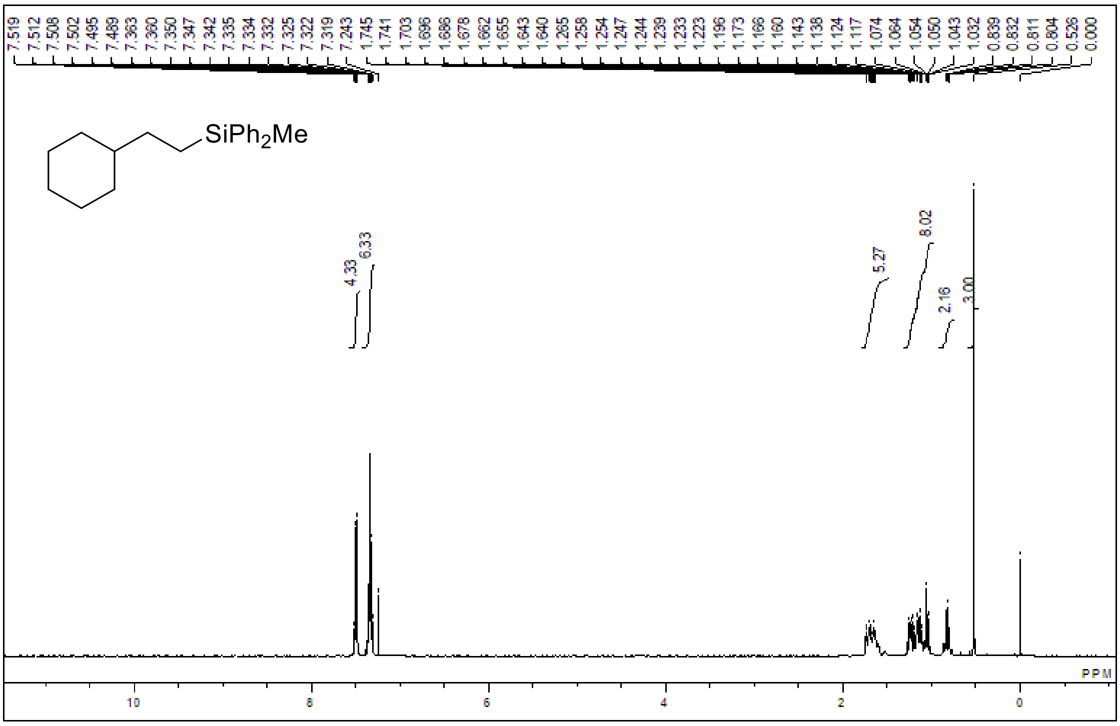

^{13}C-NMR

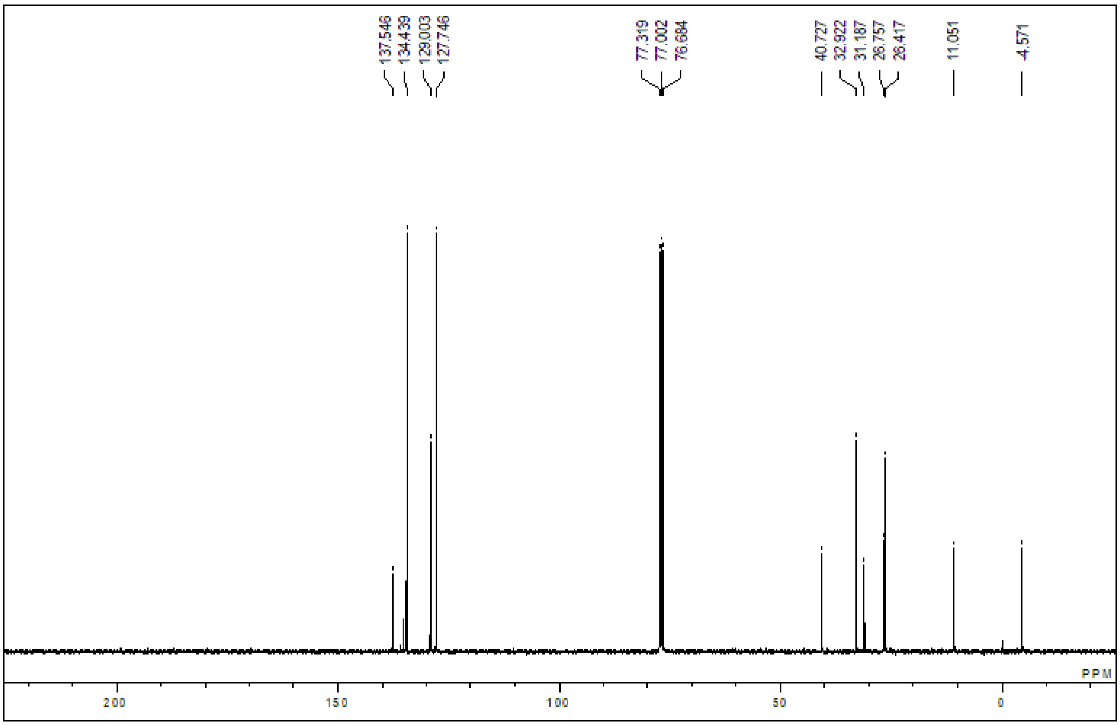

3d 1H-NMR

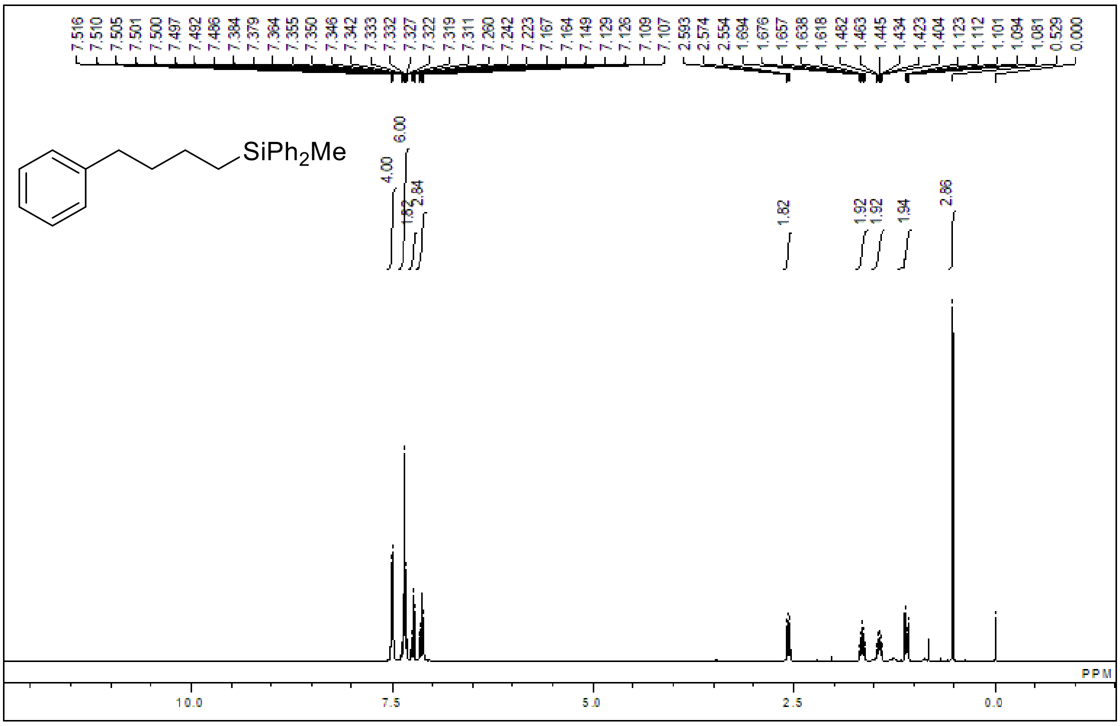

13C-NMR

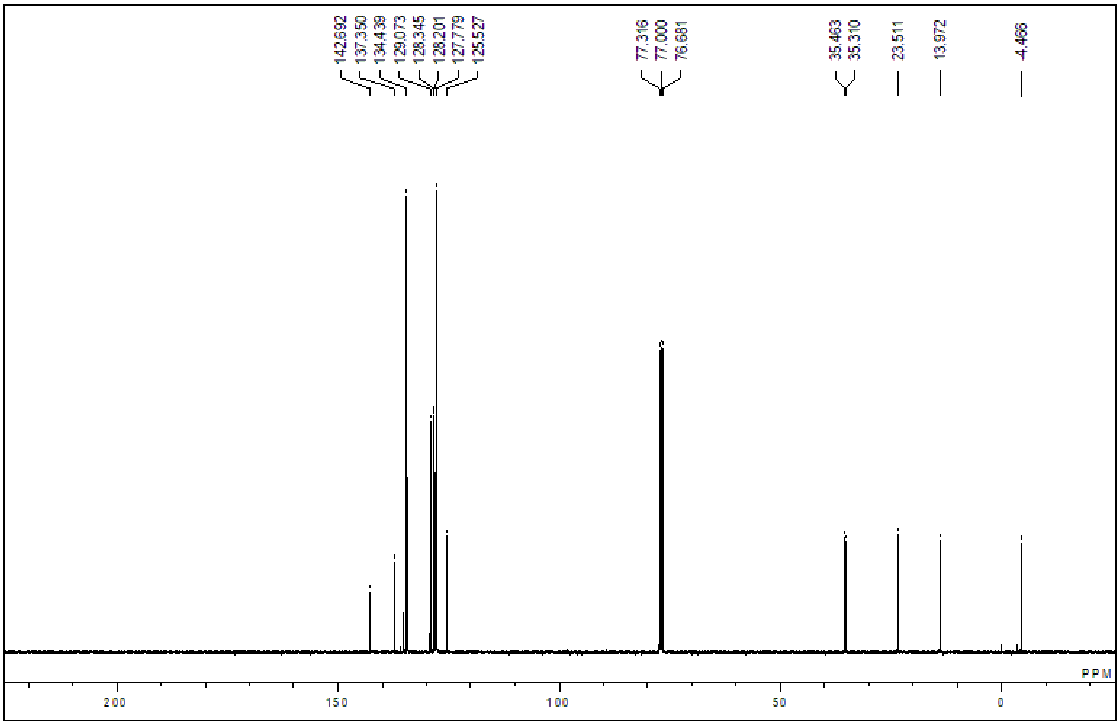

3e ^{1}H-NMR

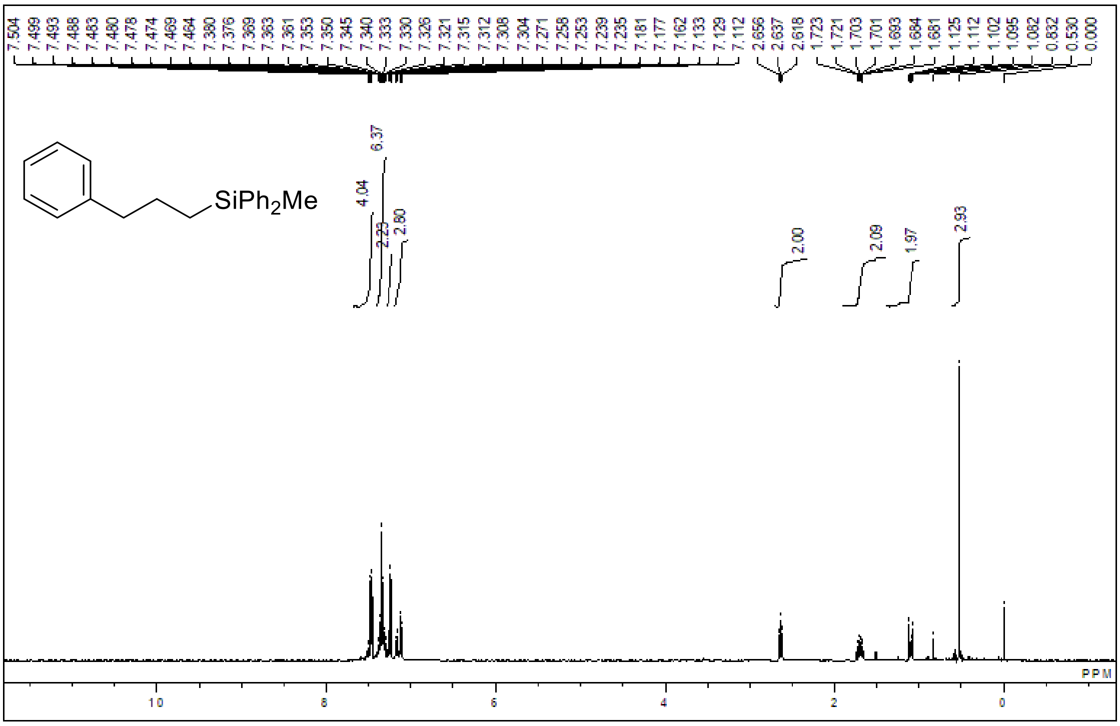

^{13}C-NMR

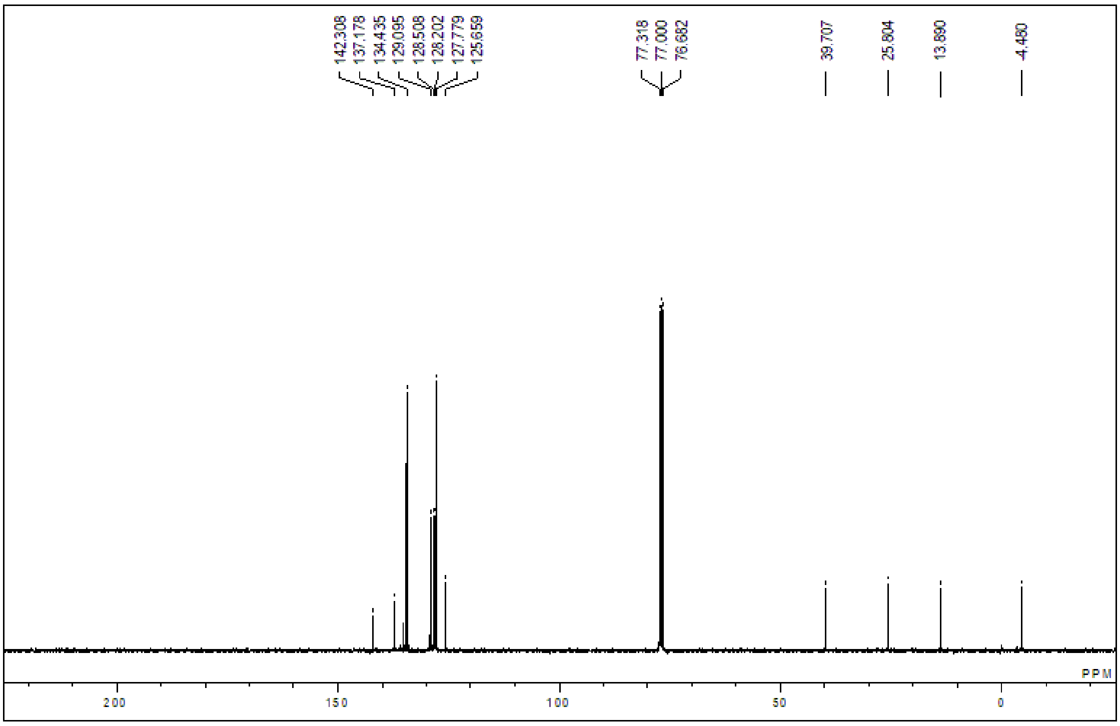

3f ^{1}H-NMR

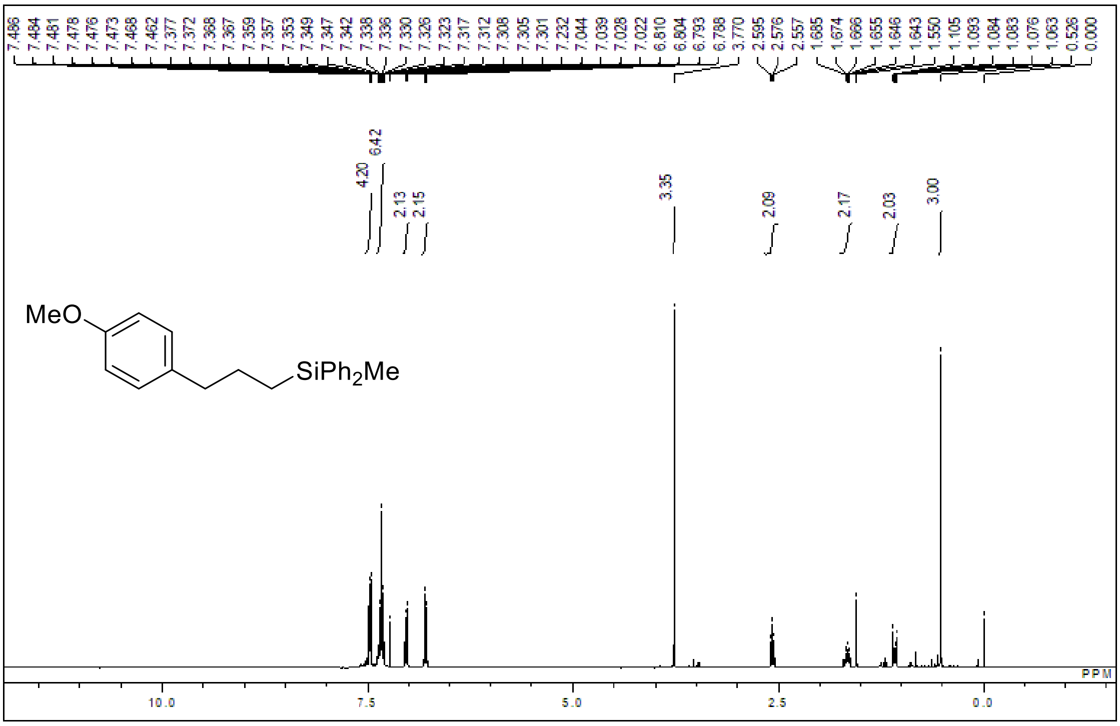

^{13}C-NMR

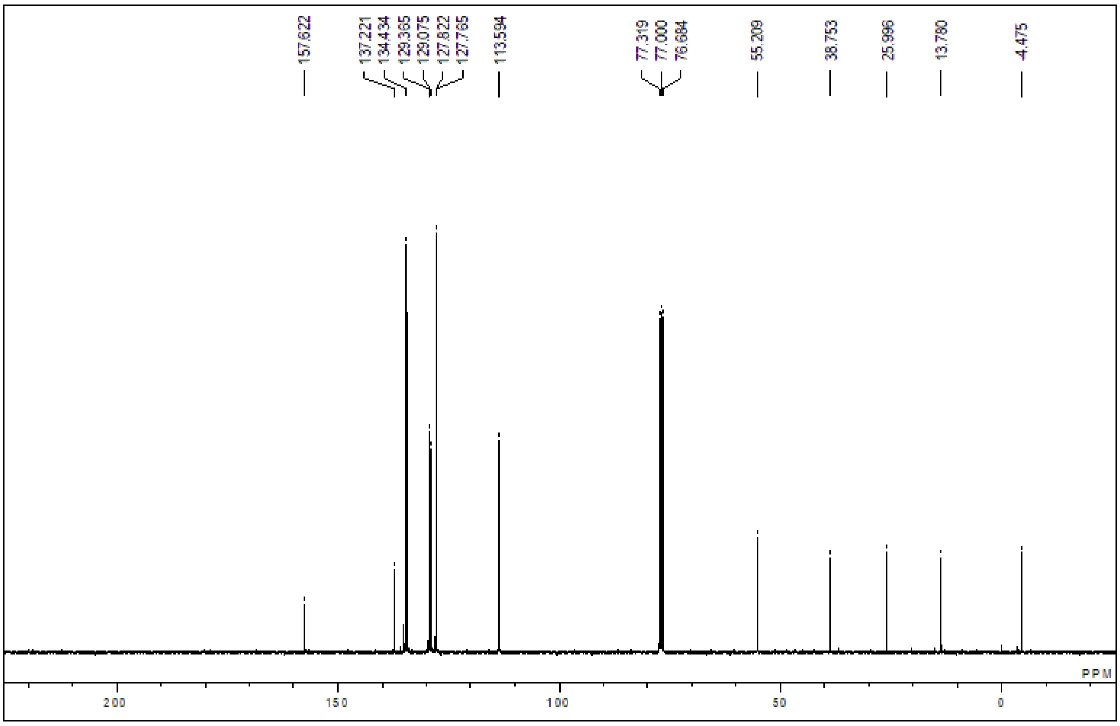

3h ^{1}H-NMR

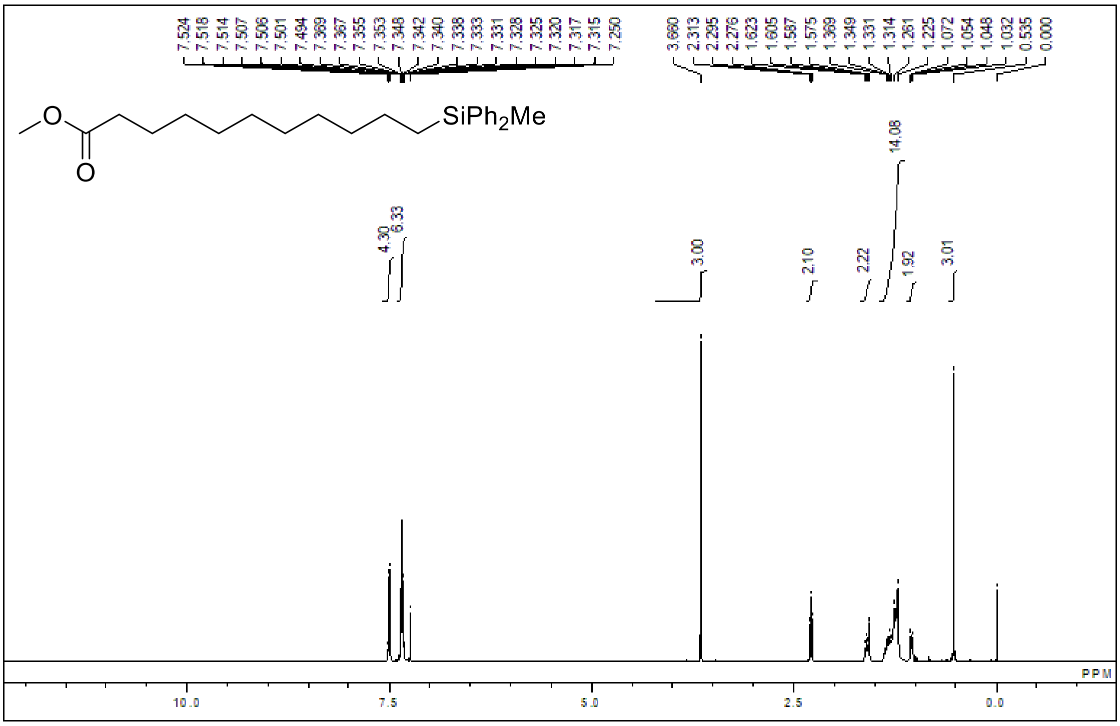

^{13}C-NMR

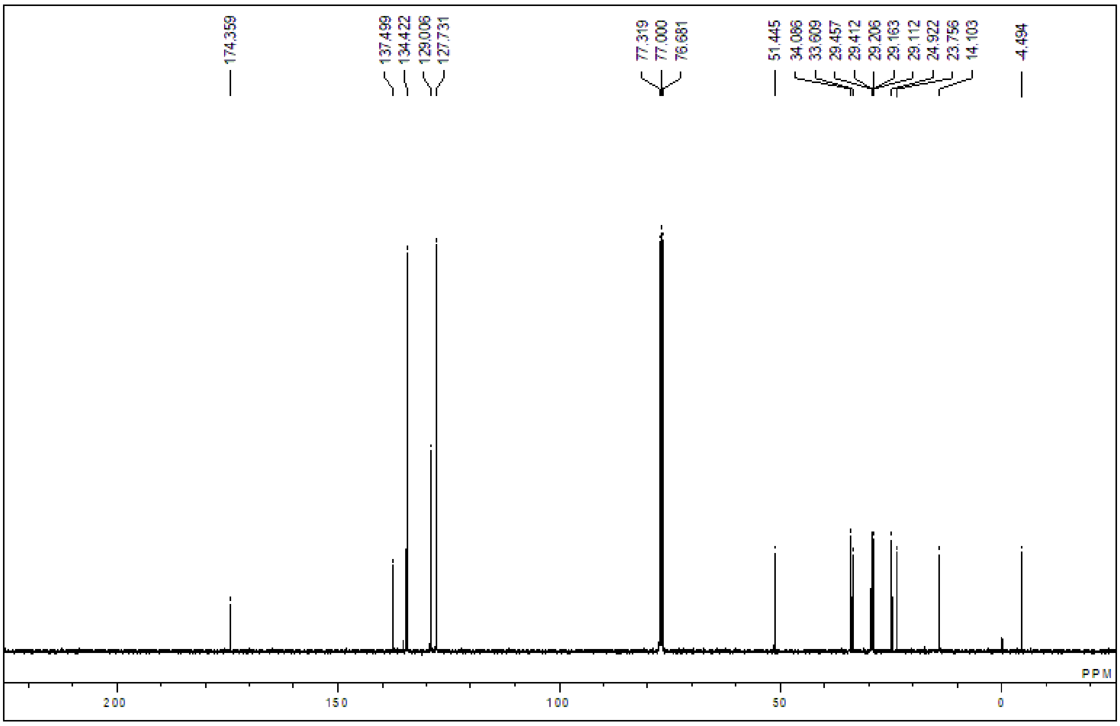

3i ¹H-NMR

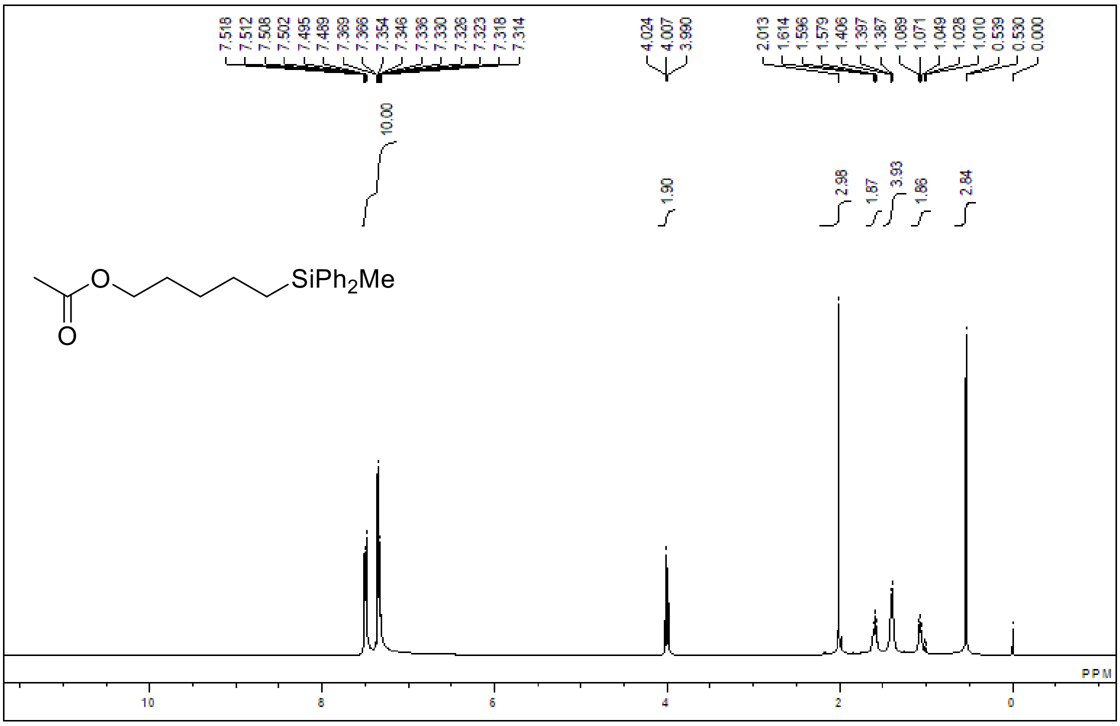

^{13}C-NMR

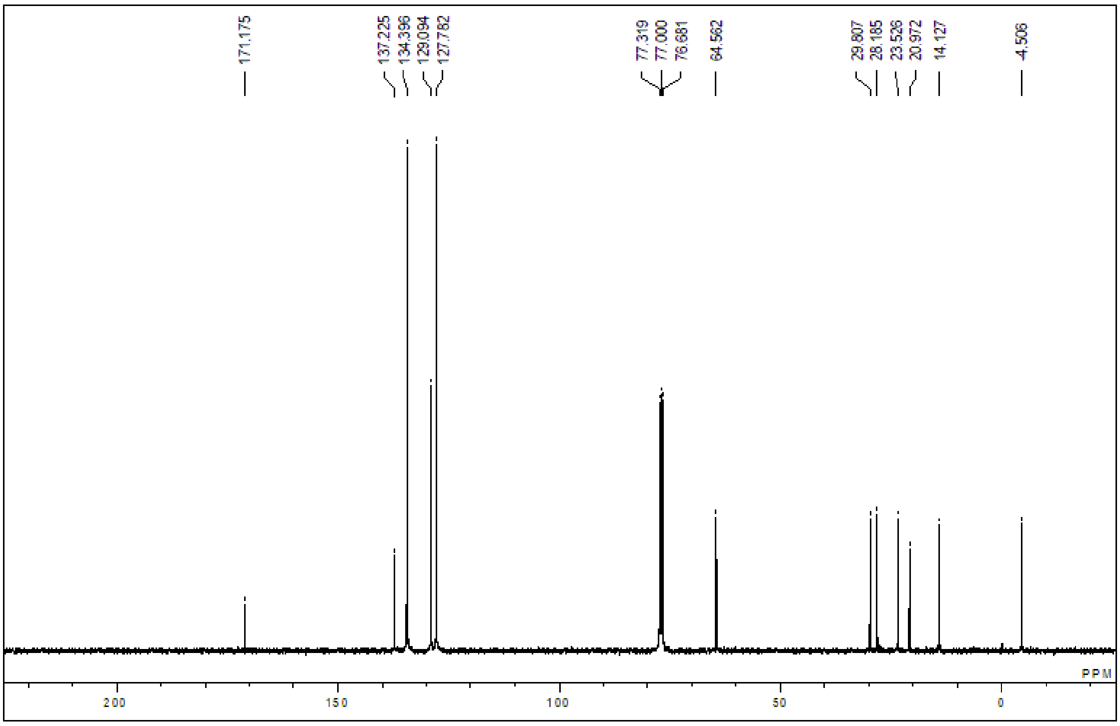

3l ^{1}H-NMR

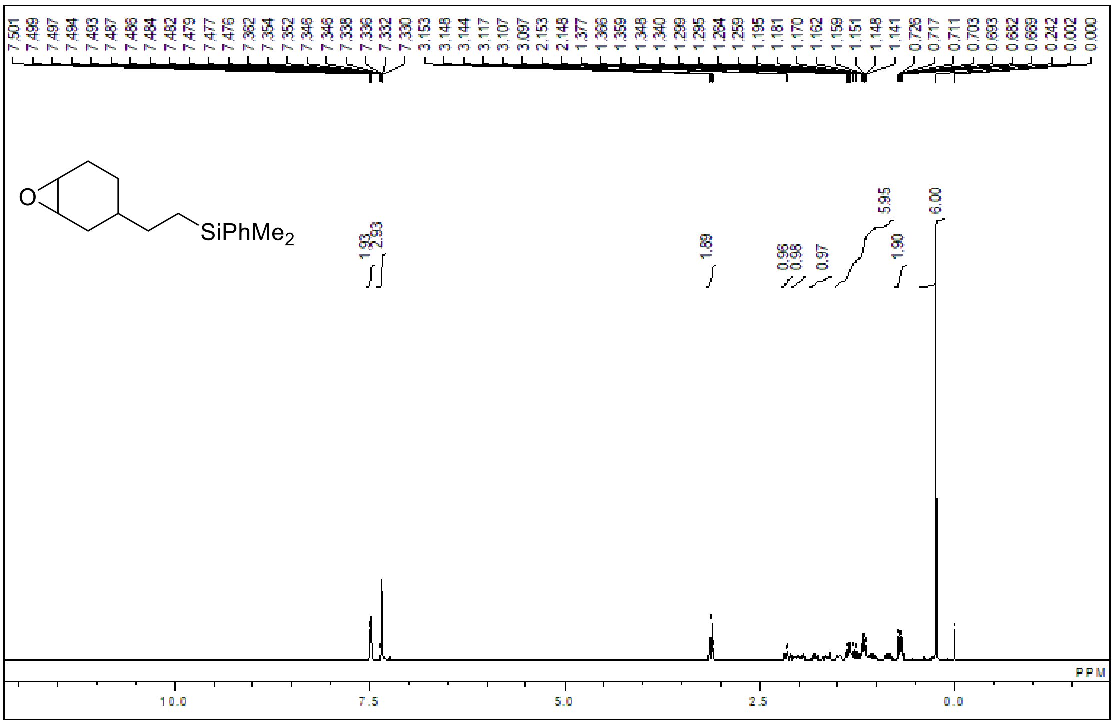

^{13}C-NMR

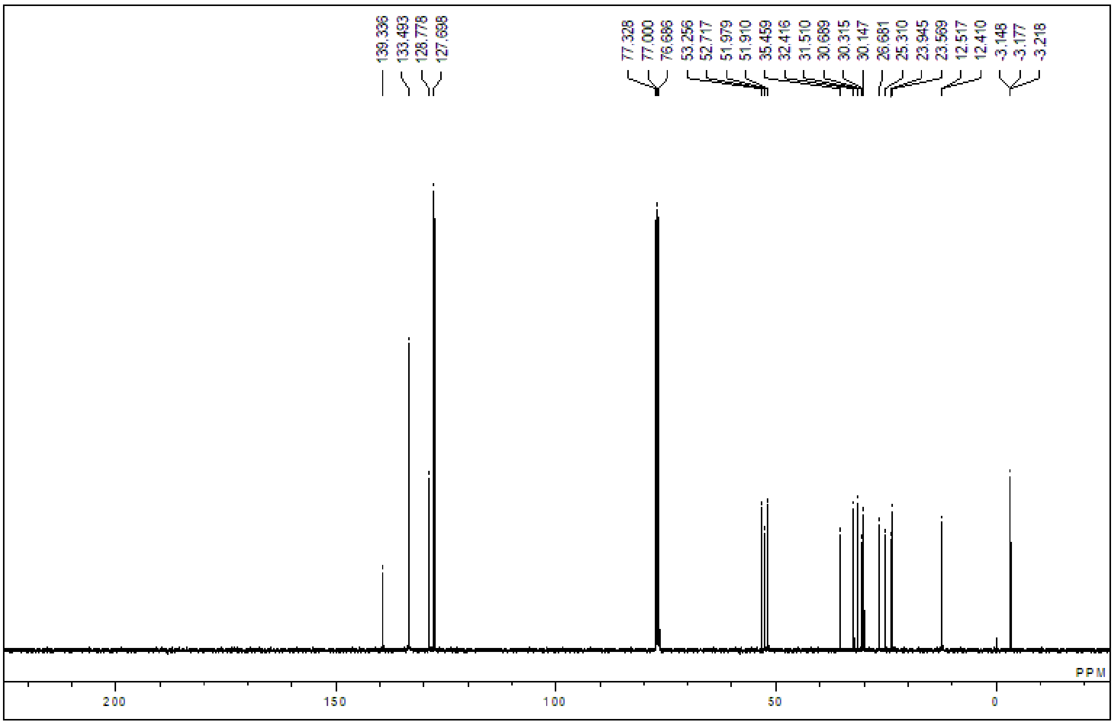

3m ^{1}H-NMR

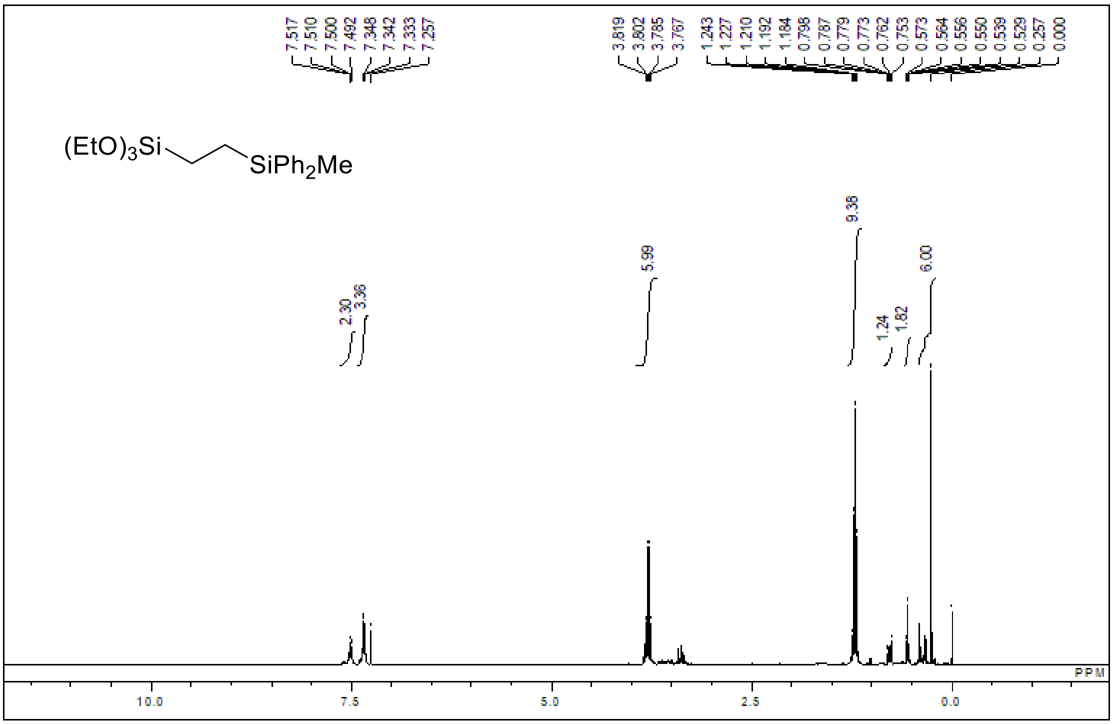

^{13}C-NMR

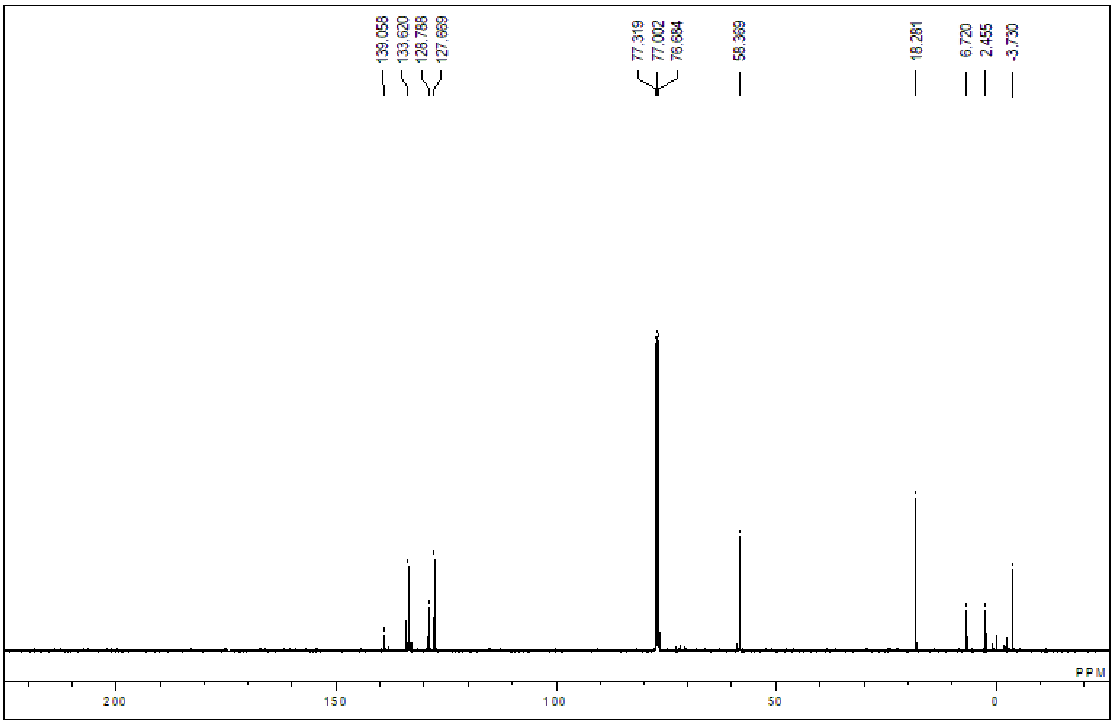

3n ^{1}H-NMR

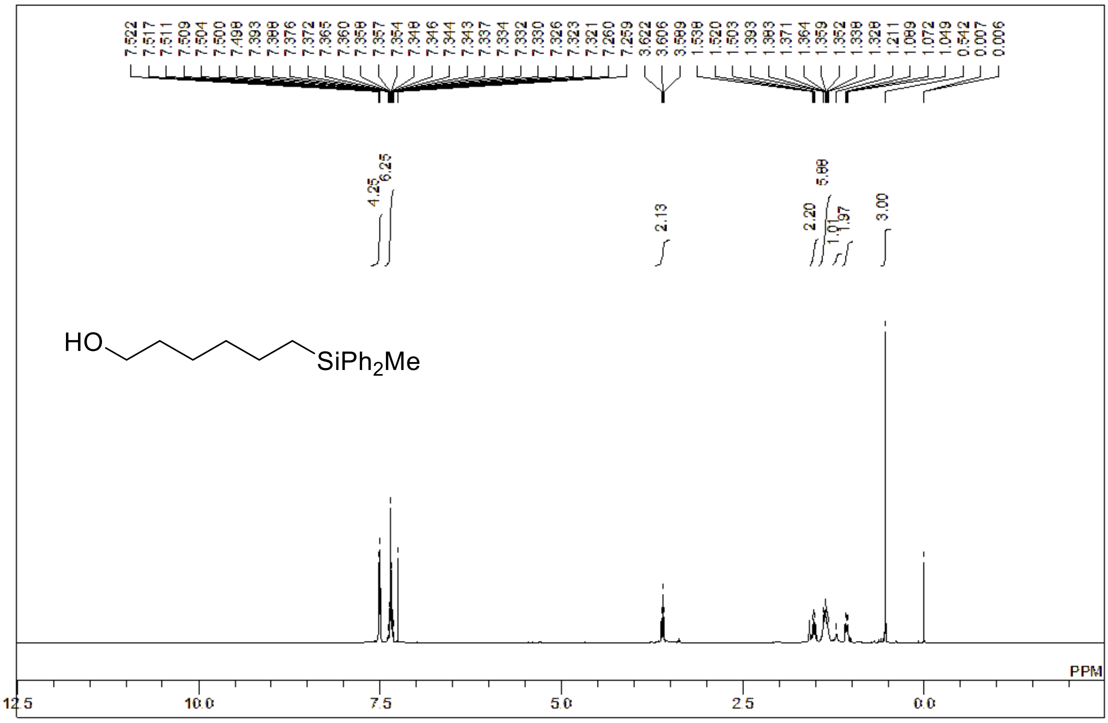

^{13}C-NMR

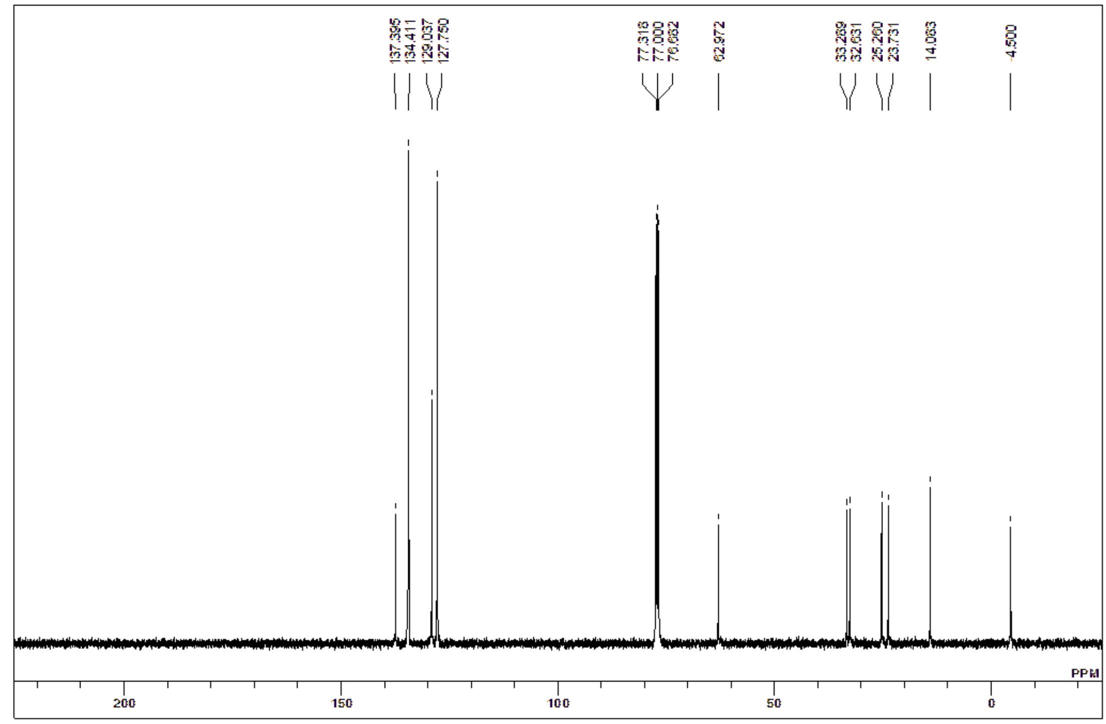

3o ^{1}H-NMR

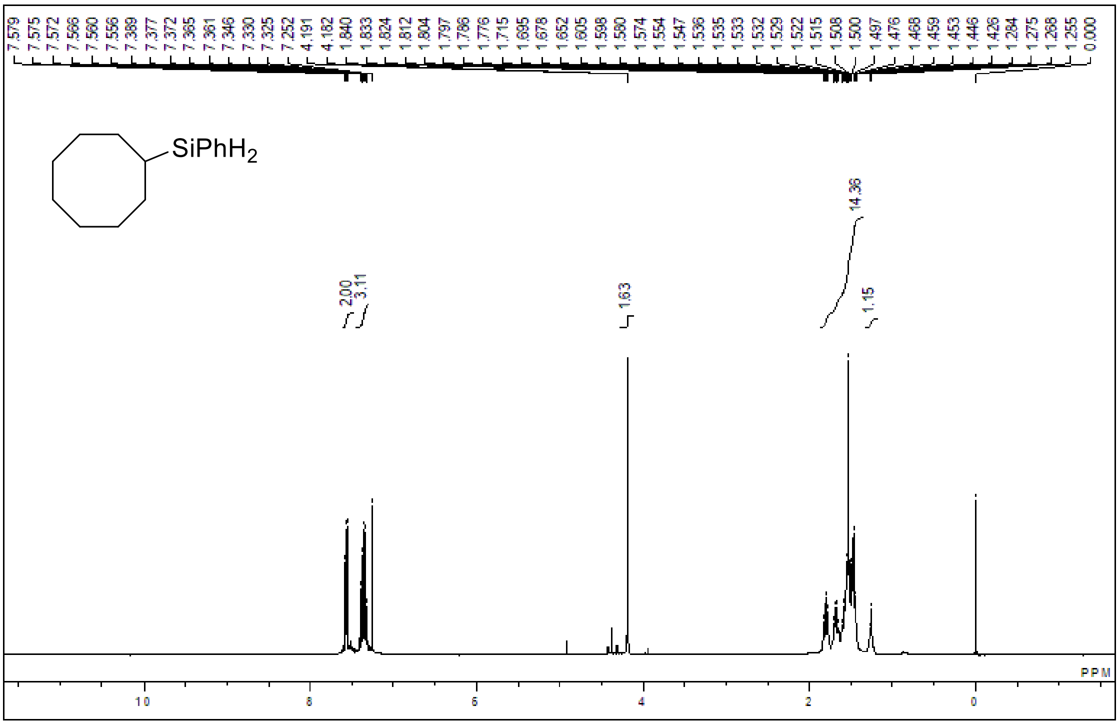

^{13}C-NMR

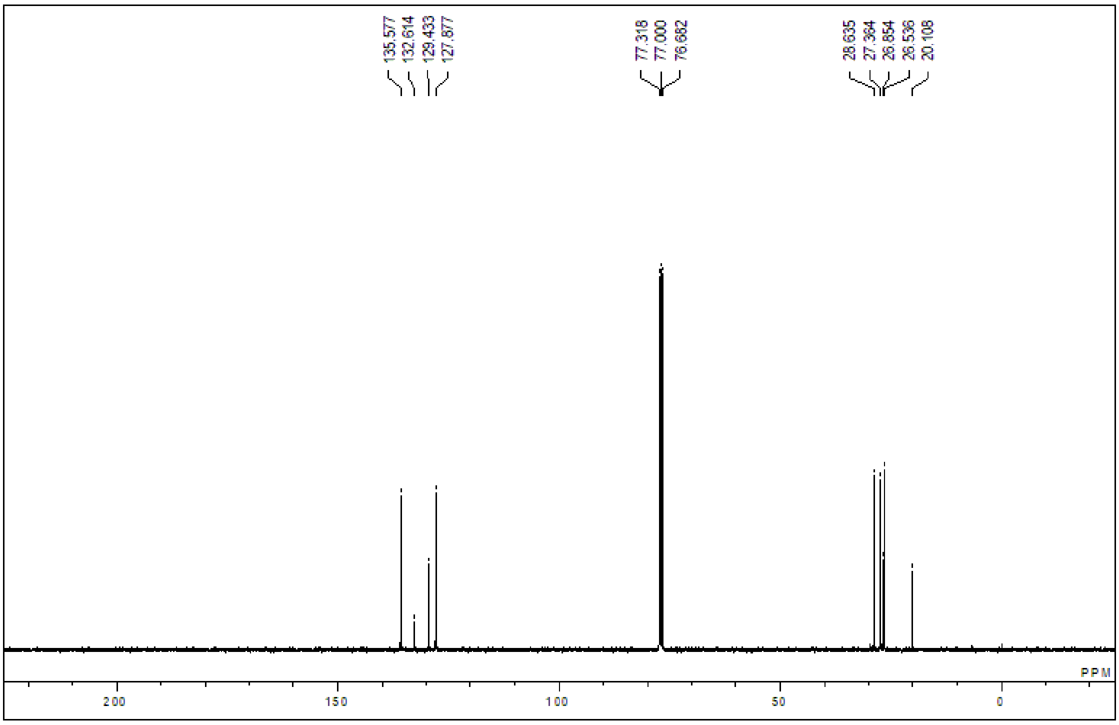

3p ^{1}H-NMR

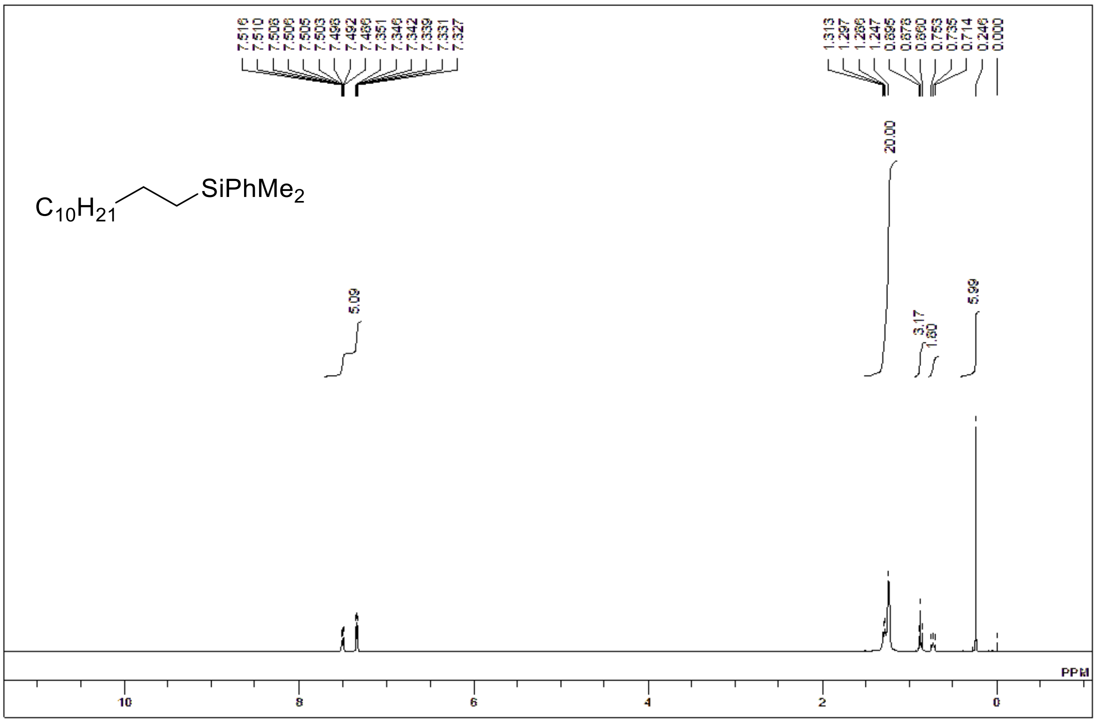

^{13}C-NMR

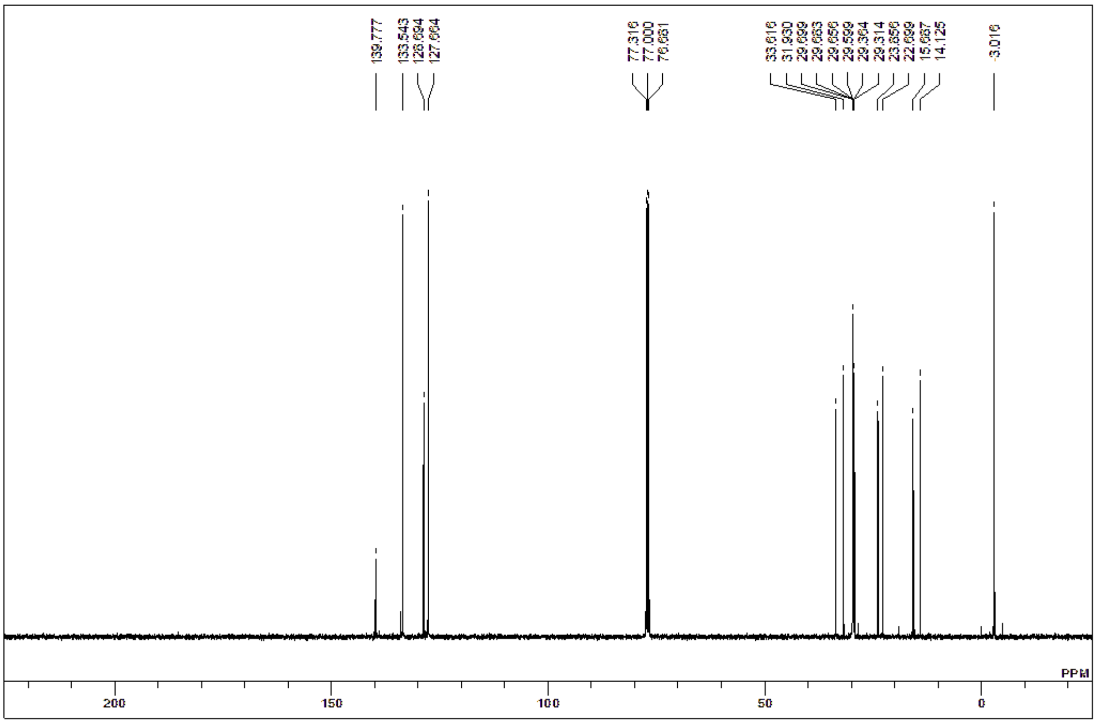

3q ^{1}H-NMR

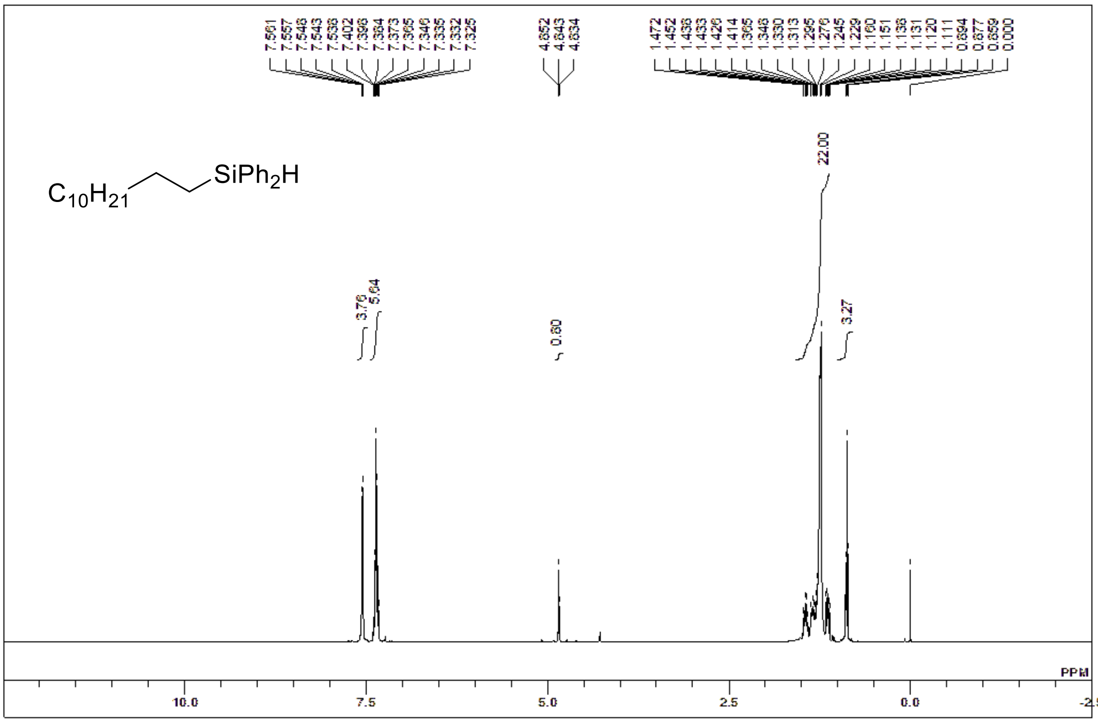

^{13}C-NMR

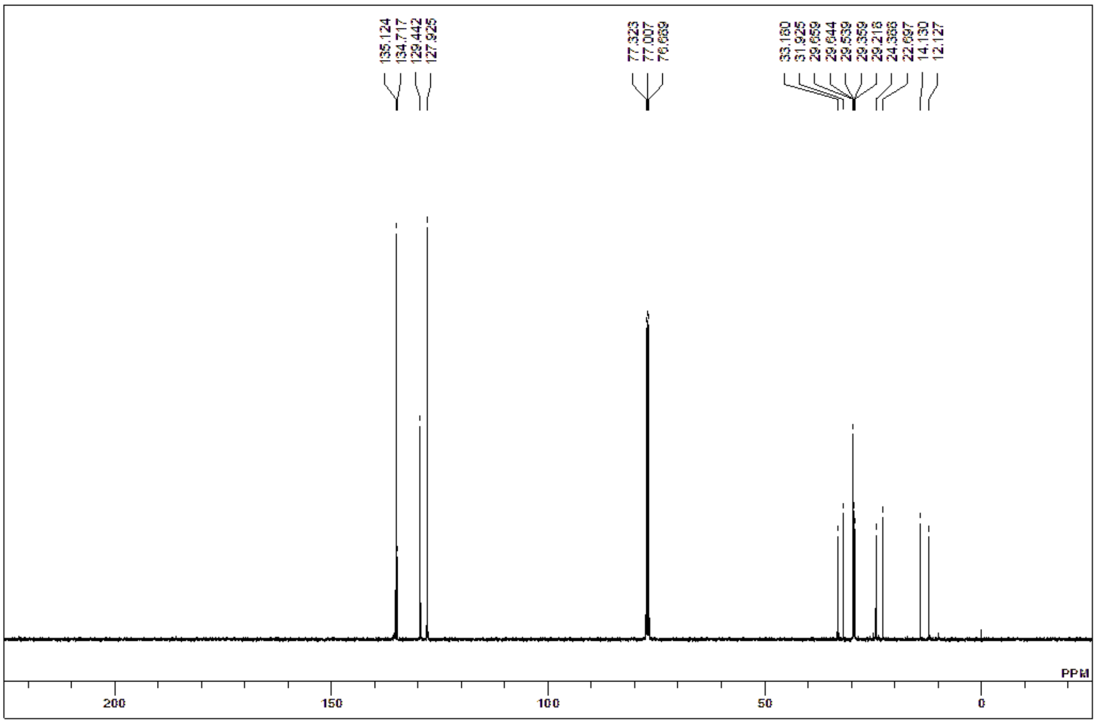

3r ^{1}H-NMR

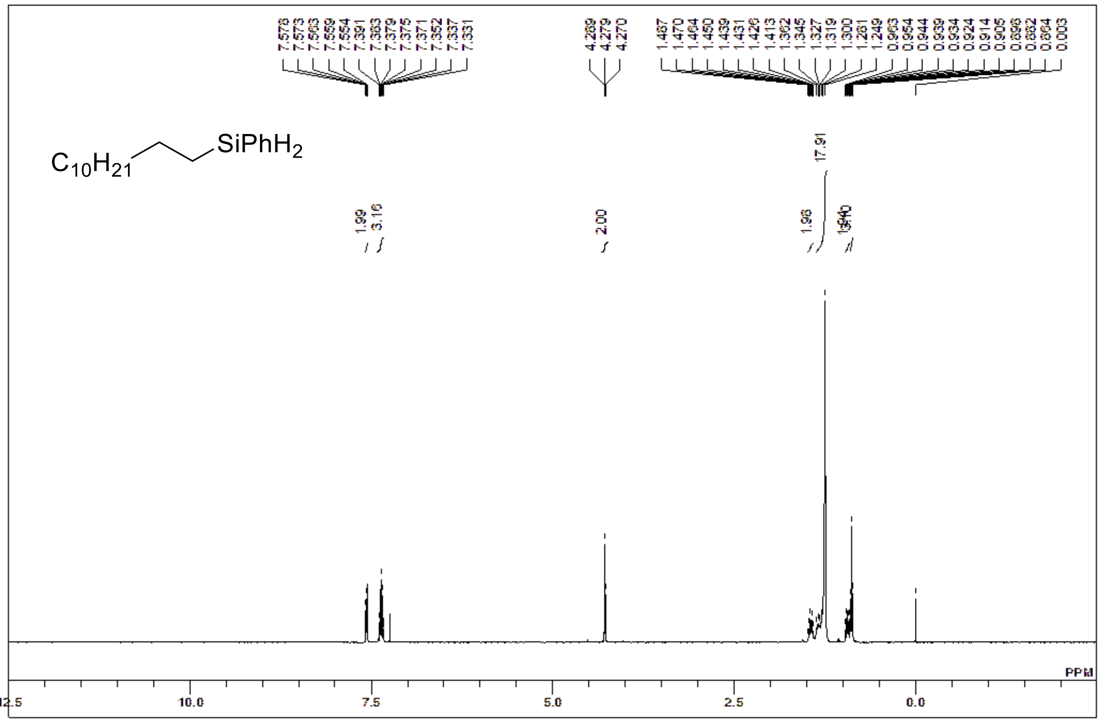

^{13}C-NMR

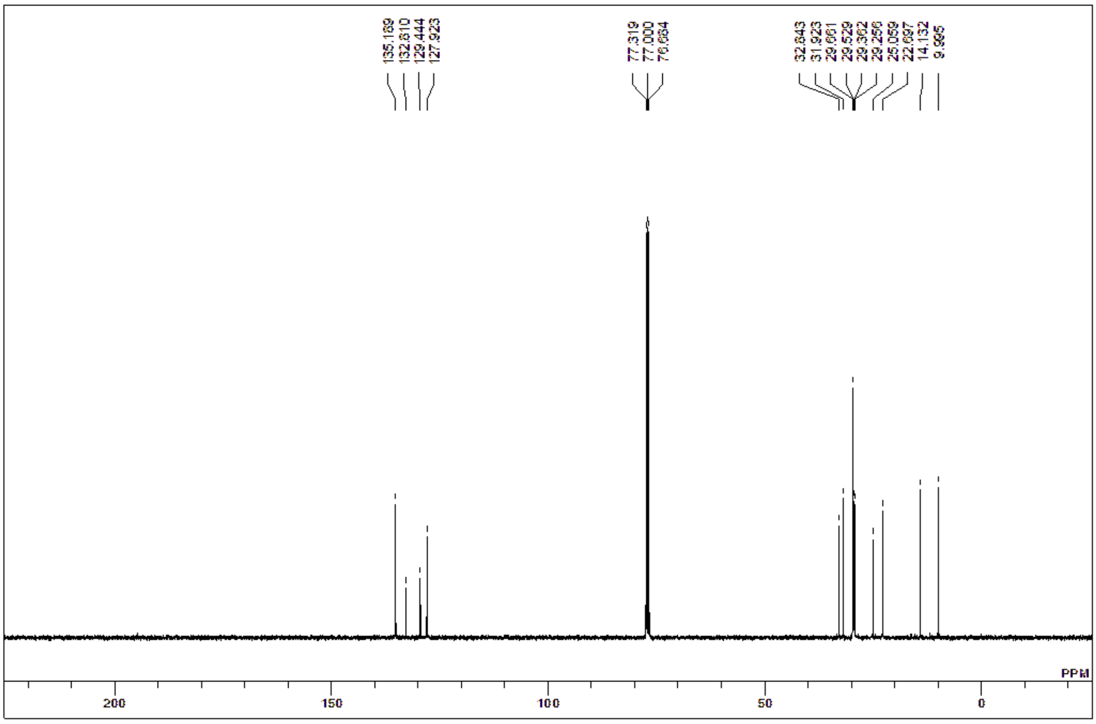

11. References

1. Miura, H.; Doi, M.; Yasui, Y.; Masaki, Y.; Nishio, H.; Shishido, T. Diverse Alkyl-Silyl Cross-Coupling via Homolysis of Unactivated C(sp^3)-O Bonds with the Cooperation of Gold Nanoparticles and Amphoteric Zirconium Oxides. *J. Am. Chem. Soc.* **2023**, *145*, 4613–4625.

2. Xu, N.-X.; Li, B.-X.; Wang, C.; Uchiyama, M. Sila- and Germacarboxylic Acids: Precursors for the Corresponding Silyl and Germyl Radicals. *Angew. Chem. Int. Ed.* **2020**, *59*, 10639–10644.

3. Hwu, J. R.; Wu, I.-F.; Shiao, S.-S.; Tseng, W. N.; Huang, J.-J.; Chen, B.-L. New Fragmentation Process in Mass Spectrometry of Carbonyl Compounds with a δ-Silyl Group. *Organometallics* **1999**, *18*, 2314–2320.

4. Zhou, R.; Goh, Y. Y.; Liu, H.; Tao, H.; Li, L.; Wu, J. Visible-Light-Mediated Metal-Free Hydrosilylation of Alkenes through Selective Hydrogen Atom Transfer for Si-H Activation. *Angew. Chem. Int. Ed.* **2017**, *56*, 16621–16625.

5. Lee, K. L. (Aminomethyl)Pyridine Complexes for the Cobalt-Catalyzed Anti-Markovnikov Hydrosilylation of Alkoxy- or Siloxy(Vinyl)Silanes with Alkoxy- or Siloxyhydrosilanes. *Angew. Chem. Int. Ed.* **2017**, *56*, 3665–3669.

6. Greenhalgh, M. D.; Frank, D. J.; Thomas, S. P. Iron-Catalysed Chemo-, Regio-, and Stereoselective Hydrosilylation of Alkenes and Alkynes Using a Bench-Stable iron(II) Pre-Catalyst. *Adv. Synth. Catal.* **2014**, *356*, 584–590.

7. Dai, Z.; Yu, Z.; Bai, Y.; Li, J.; Peng, J. Cobalt Bis(2-ethylhexanoate) and Terpyridine Derivatives as Catalysts for the Hydrosilylation of Olefins. *Appl. Organomet. Chem.* **2021**, *35*. https://doi.org/10.1002/aoc.6027.

8. Wu, X.; Ding, G.; Lu, W.; Yang, L.; Wang, J.; Zhang, Y.; Xie, X.; Zhang, Z. Nickel-Catalyzed Hydrosilylation of Terminal Alkenes with Primary Silanes via Electrophilic Silicon-Hydrogen Bond Activation. *Org. Lett.* **2021**, *23*, 1434–1439.

9. (a) Honma, T.; Oji, H; Taniguchi, Y; Ofuchi, H; Takagaki, M. Full-Automatic XAFS Measurement System of the Engineering Science Research II beamline BL14B2 at SPring-8. *AIP Conf. Proc*. **2010**, *1234*, 13–16. (b) Osaka, K.; Matsumoto, T.; Taniguchi, Y.; Inoue, D.;

Sato, M.; Sano, N. High-Throughput and Automated SAXS/USAXS Experiment for Industrial Use at BL19B2 in SPring-8. *AIP Conf. Proc.* **2016**, *1741*, 030003.

10. Ravel, B.; Newville, M. ATHENA, ARTEMIS, HEPHAESTUS : Data Analysis for X-Ray Absorption Spectroscopy Using IFEFFIT. *J. Synchrotron Rad.* **2005**, *12*, 537–541.

11. Carney, J. R.; Dillon, B. R.; Campbell, L.; Thomas, S. P. Manganese-Catalyzed Hydrofunctionalization of Alkenes. *Angew. Chem. Int. Ed.* **2018**, *57*, 10620–10624.

12. Mukhopadhyay, T. K.; Flores, M.; Groy, T. L.; Trovitch, R. J. A β-Diketiminate Manganese Catalyst for Alkene Hydrosilylation: Substrate Scope, Silicone Preparation, and Mechanistic Insight. *Chem. Sci.* **2018**, *9*, 7673–7680.

13. Yang, X.; Wang, C. Diverse Fates of β-Silyl Radical under Manganese Catalysis: Hydrosilylation and Dehydrogenative Silylation of Alkenes. *Chin. J. Chem.* **2018**, *36*, 1047–1051.

14. Dong, J.; Yuan, X.-A.; Yan, Z.; Mu, L.; Ma, J.; Zhu, C.; Xie, J. Manganese-Catalysed Divergent Silylation of Alkenes. *Nat. Chem.* **2021**, *13*, 182–190.

15. Vivien, A.; Veyre, L.; Mirgalet, R.; Camp, C.; Thieuleux, C. Mn2(CO)10 and UV Light: A Promising Combination for Regioselective Alkene Hydrosilylation at Low Temperature. *Chem. Commun.* **2022**, *58*, 4091–4094.

16. Vivien, A.; Veyre, L.; Mirgalet, R.; Camp, C.; Thieuleux, C. MnBr(CO)5: A Commercially Available Highly Active Catalyst for Olefin Hydrosilylation under Ambient Air and Green Conditions. *Green Chem.* **2023**, *25*, 7721–7728.

17. Goncharova, I. K.; Filatov, S. A.; Drozdov, A. P.; Tereshchenko, A. A.; Knyazev, P. A.; Guda, A. A.; Beletskaya, I. P.; Arzumanyan, A. V. White-Light Initiated $Mn_2(CO)_{10}$/HFIP-Catalyzed Anti-Markovnikov Hydrosilylation of Alkenes. *J. Catal.* **2024**, *429*, 115269.
